# Binding and functional profiling of antibody mutants guides selection of optimal candidates as antibody drug conjugates

**DOI:** 10.1371/journal.pone.0226593

**Published:** 2019-12-31

**Authors:** John C. Zwaagstra, Traian Sulea, Jason Baardsnes, Stevo Radinovic, Yuneivy Cepero-Donates, Alma Robert, Maureen D. O’Connor-McCourt, Ilia A. Tikhomirov, Maria Luz. Jaramillo

**Affiliations:** 1 Human Health Therapeutics Research Centre, National Research Council Canada, Montreal, Quebec, Canada; 2 Forbius, Montreal, Quebec, Canada; Weizmann Institute of Science, ISRAEL

## Abstract

An increasingly appreciated conundrum in the discovery of antibody drug conjugates (ADCs) is that an antibody that was selected primarily for strong binding to its cancer target may not serve as an optimal ADC. In this study, we performed mechanistic cell-based experiments to determine the correlation between antibody affinity, avidity, internalization and ADC efficacy. We used structure-guided design to assemble a panel of antibody mutants with predicted Her2 affinities ranging from higher to lower relative to the parent antibody, Herceptin. These antibodies were ranked for binding via SPR and via flow-cytometry on high-Her2 SKOV3 cells and low-Her2 MCF7 cells, the latter acting as a surrogate for low-Her2 normal cells. A subpanel of variants, representative of different Her2-binding affinities (2 strong, 2 moderate and 3 weak), were further screened via high-content imaging for internalization efficacies in high versus low-Her2 cells. Finally, these antibodies were evaluated in ADC cytotoxicity screening assays (using DM1 and MMAE secondary antibodies) and as antibody-drug conjugates (DM1 and PNU159682). Our results identified specific but weak Her2-binding variants as optimal candidates for developing DM1 and PNU ADCs since they exhibited high potencies (low to sub-nM) in high-Her2 SKOV3 cells and low toxicities in low-Her2 cells. The 2 strong-affinity variants were highly potent in SKOV3 cells but also showed significant toxicities in low-Her2 cells and therefore are predicted to be toxic in normal tissues. Our findings show that pharmacological profiling of an antibody library in multiple binding and functional assays allows for selection of optimal ADCs.

## Introduction

The efficacy of an ADC relies on numerous factors including characteristics of the ADC itself as well as the biologic features of the target and tumor cell. Key factors include the binding affinity of the antibody, internalization and trafficking of the ADC, transport and release of the drug from the endocytosed compartment to the cytosol, the cytosolic concentration and mode of action of the drug [1, 2].

The commonly used payload drugs in ADCs are highly potent cytotoxic molecules. Some examples are derivatives of calicheamicin (DNA targeting) and microtubule inhibitors such as maytansine (i.e. DM1 and DM4) and auristatins (i.e. monomethyl auristatin E, MMAE). Off-target platform-related toxicities are frequently seen for ADCs that incorporate these potent drugs, largely due to premature drug release in the blood stream and subsequent systemic exposure. The typical clinical manifestations seen with conventional ADCs containing auristatins and maytansines are bone marrow toxicity and peripheral neuropathy [3, 4]. Furthermore, on-target (off-tumor) toxicity, due to specific binding of an ADC to normal tissues expressing low levels of target antigen, presents another formidable challenge and is not always predictable [5, 6]. One example is an early clinical trial with AML (acute myeloid leukemia) patients treated with an anti-CD33 calicheamicin (gentuzumab ozogamicin, aka Mylotarg) [7]. Severe bone marrow hypoplasia and myelosuppression (neutropenia) were observed and one patient died. This was attributed to on-target binding of CD33 on the surface of myeloid progenitor cells. Other examples are; bivatuzumab mertansine, which induced severe skin toxicities due to binding to CD44v6 target on normal keratinocytes, and MEDI-547 (anti-Ephrin A2 receptor antibody-auristatin) which caused significant bleeding and coagulation, requiring termination of clinical development of these ADCs [8, 9]. Toxicity concerns are further compounded when using more potent anthracycline-based drugs such as PNU159682 which intercalate DNA and can target non-dividing cells [10]. Hence, it is important to minimize these serious side-effects and find the right balance between ADC potency and toxicity in order to achieve the optimal therapeutic window.

Recent seminal reports have demonstrated that there is a significant interplay between antibody affinity, target density/avidity and antibody valency. Harms et al [11] used kinetic computational models and flow cytometry measurements to evaluate the effect of different EGFR target densities on the binding of anti-EGFR Fab and full-size antibodies (FSAs) on cells. Strength of binding was shown to be strongly augmented by high target density/avidity. Another group examined a panel of EGFR single-chain Fv (scFv) and Fab antibody fragments that bind to the same EGFR epitope with varying affinities [12]. Binding measurements of the respective monovalent scFv or Fab versus bivalent FSA versions to EGFR-expressing cells showed a dramatic increase in apparent binding affinities (by 100- to 1000-fold) for the weak-affinity Fabs/scFvs when converted to bivalent FSA formats. Large-magnitude changes in apparent affinity due to avidity effects were observed in another study that tested the binding of affinity variants of an anti-Her2 monoclonal antibody to Her2-expressing cancer cells [13]. Beyond cell surface binding, this above mentioned study also showed that both affinity and rate of Her2 internalization dictate the intracellular distribution of anti-Her2 antibodies, thus determining their catabolic fate.

More recently, target avidity was exploited in terms of developing better T cell-dependent bispecific (TDB) antibodies. In this study, the strong contribution of target avidity for promoting Her2 tumor selectivity was shown using anti-Her2/CD3 bispecific TBD constructs having one arm comprised of a bivalent-Fab with a range of monovalent Her2 affinities [14]. Low affinity TDBs showed cell surface binding and enabled selective killing of high-Her2 tumor cells (SKBr3) while exhibiting no binding to, or killing of, low-Her2 cells (MCF7). High affinity TBDs readily killed both cell types.

The above studies, which dealt with non-conjugated antibodies as therapeutics, convey the importance of evaluating a wide affinity range and exploiting differential target density/avidity effects at the cell surface. Considering that ADC targets are typically chosen due to differential expression in tumor versus normal tissue, it follows that similar assessments should be incorporated upfront in any procedure for screening antibodies for their potential as ADCs. Since high target density on the surface of a cancer cell has been shown to promote binding of low-affinity antibodies, it is apparent that weaker antibodies should not be de-prioritized in the early-phase ADC selection processes.

We proposed to develop a predictive *in vitro* screening approach to assess antibodies with varying affinities for their potential as ADCs. Our method begins with the classification of antibodies based on binding affinities and target avidity at the cell surface. This is followed by assessments focused on the intracellular mechanisms required for ADC activities. Notably, after binding to the cell surface, an ADC must internalize efficiently and traffic to the appropriate cellular compartment (e.g. endosome, lysosome) for processing and drug release. Accordingly, in this study candidate ADC antibodies were evaluated for internalization efficiency, intracellular accumulation and cytotoxic potency.

As proof-of-principle, we applied our screening procedure to a rationally designed panel of Herceptin variants ranging from high to low affinity for Her2. These antibodies were ranked based on relative binding affinity to cells having high or low-Her2 densities and were classified as strong, moderate or weak binders. Representative variants from each group were further tested for internalization, followed by cytotoxicity testing with three drugs; DM1, MMAE and PNU159682 (PNU). Our results demonstrate that weak binding antibodies, with affinity *K*_D_s of ~70 nM or more, can exhibit significant ADC potencies in high-Her2 cancer cells while having low toxicities in low-Her2 cells. In contrast, strong binding Her2 antibodies with affinity *K*_D_s of ~1 nM or less exhibit high toxicities on both high- and low-Her2 cells, which may be disadvantageous particularly in the context of very potent payload drugs such as PNU.

## Results

### Design of Herceptin Fab variants with a wide range of antigen binding affinities

We set out to assemble a panel of about 20 mutational variants of Herceptin Fab that would cover over 4 orders of magnitude in antigen binding affinity as indicated by the monovalent dissociation constant, *K*_D_. It proved to be a challenge to find mutations that increased binding affinity above that naturally occurring with Herceptin (*K*_D_ of 0.5 nM) [15]. However, we identified a few variants with higher affinity and a large number of variants with a wide range of weakened antigen binding affinities. Together, these variants enabled us to examine the premise that a weakened monovalent binding affinity, coupled with the avidity potential of bivalent mAbs, would confer preferential targeting of cells overexpressing the antigen and thus lead to reduced ADC toxicity on cells expressing a low level of antigen.

The panel included Herceptin Fab (termed Parent 1) and derived variants generated by structure-based computational design. As well, a previously characterized Herceptin-derived variant, bH1 Fab, which is a dual-action Fab (DAF) that binds both Her2 and VEGF, was included. This phage-display-derived bH1 Fab, which we term Parent 2, is a variant of Herceptin with a 4-amino-acid residues insertion in the CDR-L1 loop as well as a few point mutations. It has a binding affinity to Her2 of 20 nM (40-fold weaker relative to Herceptin Fab) [15].

**Fig 1:**
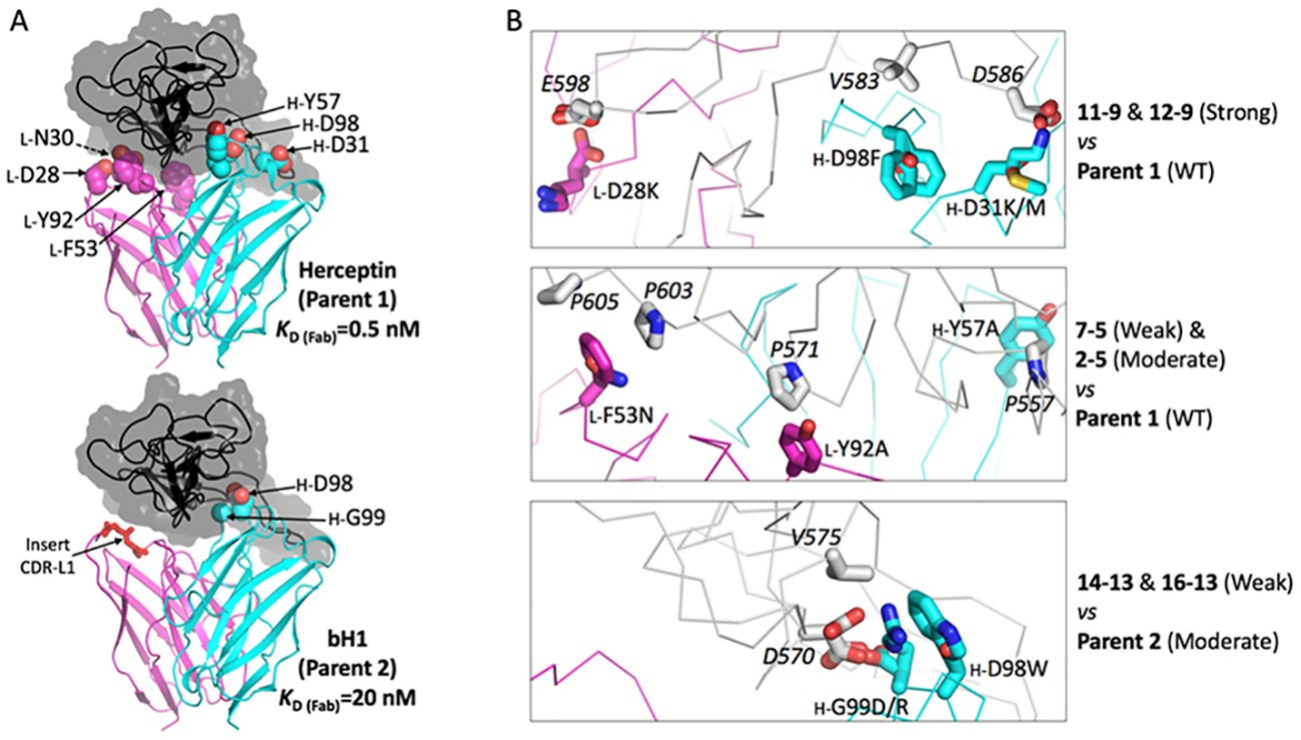
Structure-based computational design of a Fab mutant set with varied binding affinity to Her2 ectodomain. (**A**) Structural overview of the parent antibodies Herceptin and bH1 binding the same epitope of the Her2 antigen. Crystal structures with PDB codes 1N8Z and 3BE1 are used for Herceptin-Fab/Her2 and bH1-Fab/Her2 complexes, respectively (see Materials and methods). Measured Her2 binding affinities of the corresponding Fab fragments are from Bostrom et al. [15] Only the antigen-binding Fv domains of the antibodies are shown as ribbons colored cyan and magenta for the heavy and light chains, respectively. The epitope of the Her2 antigen is rendered as black ribbon inside a translucent gray molecular surface. Mutated positions of the antibodies are shown as CPK models and labeled. The position of a four-residue insert in the CDR-L1 of bH1 relative to Herceptin is also indicated. (**B**) Molecular details of interactions at the mutated positions of selected antibody mutants. Each sub-panel includes an overlay of molecular models for several antibodies (mutants vs. parent). Mutated antibody residues are labeled and shown as sticks with C atoms colored cyan and magenta for the heavy and light chains, respectively. Her2 residues interacting with mutated Fab positions are shown as stick with white C atoms and labeled in italics.

Computational design was initiated with the Her2-complexed crystal structures of Parent 1 and Parent 2 aiming to generate variants with altered antigen binding affinities that can be binned into 4 classes: Strong, Wild-Type-like (WT), Moderate and Weak. In this classification, Parent 1 belongs to the WT class whereas Parent 2 (40-fold increase in *K*_D_, equivalent to 2.15 kcal/mol increase in binding free energy) is placed in the Moderate class. Table 1 lists the predicted binding affinity ranking and classes for an additional 18 mutants derived from Parent 1 (15 variants) and Parent 2 (3 variants). According to these structure-based computational predictions, the distribution in the Strong, WT, Moderate and Weak classes is 4, 4, 5 and 7 variants, respectively. This meets the design criteria for the Fab panel. No significant destabilization of Fab folding was predicted for any of the designed variants. One or more of 7 amino-acid positions located in the CDR loops were utilized to derive the variants originating from Parent 1 (Fig 1A upper panel) and 2 positions for the variants derived from Parent 2 (Fig 1A bottom panel). Overall, mutagenesis targeted only 4 sites in either the heavy chain (D31, Y57, D98 and G99) or the light chain (D28, N30, F53 and Y92) of the Fab.

**Table 1:** Structure-based computational design of the Fab-mutant set with varied binding affinities to Her2 ectodomain.

ID	Mutations	Relative binding affinity (ѦѦ*G*_bind_) ^b^			Consensus score ^c^	Predicted class ^d^
		FoldX	Rosetta	SIE-Scwrl		
***SUBSET 1***						
12–9	*H*-D98F, *H*-D31K, *L*-D28K	-1.60	-0.92	-0.83	-1.10	Strong
11–9	*H*-D98F, *H*-D31M, *L*-D28K	-1.12	-0.90	-0.61	-0.86	Strong
11–1	*H*-D98F, *H*-D31K	-0.96	-0.44	-0.81	-0.79	Strong
12–1	*H*-D98F, *H*-D31M	-0.60	-0.42	-0.60	-0.59	Strong
10–1	*H*-D98F	-0.23	-0.48	-0.26	-0.33	WT
2–9	*L*-D28K	-0.43	-0.47	0.06	-0.21	WT
**2–1**	**Parent 1 (Herceptin-Fab); *K***_**D**_ **= 0.5 nM** ^a^	0.00	0.00	0.00	0.00	WT
2–4	*L*-Y92A	0.38	1.73	0.70	0.97	WT
7–1	*H*-Y57A	2.07	1.07	0.49	1.07	Moderate
2–3	*L*-F53N	1.92	1.88	0.39	1.22	Moderate
2–6	*L*-N30D	3.09	0.86	1.57	1.84	Moderate
2–5	*L*-F53N, *L*-Y92A	2.07	3.61	0.78	1.97	Moderate
7–4	*H*-Y57A, *L*-Y92A	2.26	2.81	1.69	2.27	Weak
7–3	*H*-Y57A, *L*-F53N	3.98	2.95	0.87	2.28	Weak
7–6	*H*-Y57A, *L*-N30D	5.18	1.93	2.06	2.91	Weak
7–5	*H*-Y57A, *L*-F53N, *L*-Y92A	4.30	4.68	1.65	3.28	Weak
***SUBSET 2***						
**2–13**	**Parent 2 (bH1-Fab); *K***_**D**_ **= 20 nM** ^a^	0.00	0.00	0.00	0.00	Moderate
15–13	*H*-G99D	7.71	2.11	0.24	1.37	Weak
16–13	*H*-G99R	9.44	13.73	-0.93	1.47	Weak
14–13	*H*-D98W, *H*-G99D	10.19	1.77	0.31	1.71	Weak

^a^ Data from Ref. [15]

^b^ Calculated data with indicated methods, in kcal/mol units.

^c^ Average of Z-scores for indicated methods, relative to respective Parent, in standard deviation (SD) units (see Methods section).

^d^ Consensus score increments between classes are of +1 SD, except for the Strong class with a -1/2 SD increment, relative to respective Parent.

Various molecular mechanisms were engaged in the design of mutants in these affinity classes. The affinity-improved variants in the Strong class, which originated from an ADAPT affinity maturation campaign of Parent 1 [16], are double- and triple-point mutants that eliminate electrostatic repulsions with D586 and E598 of Her2 (Fig 1B upper panel) by replacing some negatively charged residues of Parent 1 with non-polar or positively charged residues (*L*-D28K, *H*-D31K/M, *H*-D98F). Because single-point mutants at these Fab positions led to smaller affinity-strengthening effects they were placed in the WT class (Table 1).

The eight affinity-weakened variants in the Moderate and Weak classes derived from Parent 1 were based on a completely distinct strategy (Fig 1B middle panel). This relied on removing a few favorable non-polar packing contacts between one or more aromatic residues of the Fab and several Pro residues of Her2 (P557, P571, P603 and P605). Size-reduction mutations at three Fab positions (*L*-F53N, *L*-Y92A, *H*-Y57A) led to mild reductions of Fab binding affinity with this Pro-rich region of the antigen’s protein-protein interface. Double- and triple-point combinations of these mutations had larger effects than individual single-point mutants (Table 1).

The three affinity-weakened variants in the Weak class derived from Parent 2 (itself in the Moderate class, see Table 1) also implicated a distinct mechanism. For these variants, a combination of size increase in the CDR-H3 loop of the Fab (*H*-G99D/R, *H*-D98W) and modulation of electrostatic interactions with the charged residue D570 of Her2 (Fig 1B bottom panel) were utilized in order to only mildly weaken the affinity of Parent 2.

### SPR measurement of Her2 binding affinities and ranking of Herceptin full-size antibody variants

**Fig 2:**
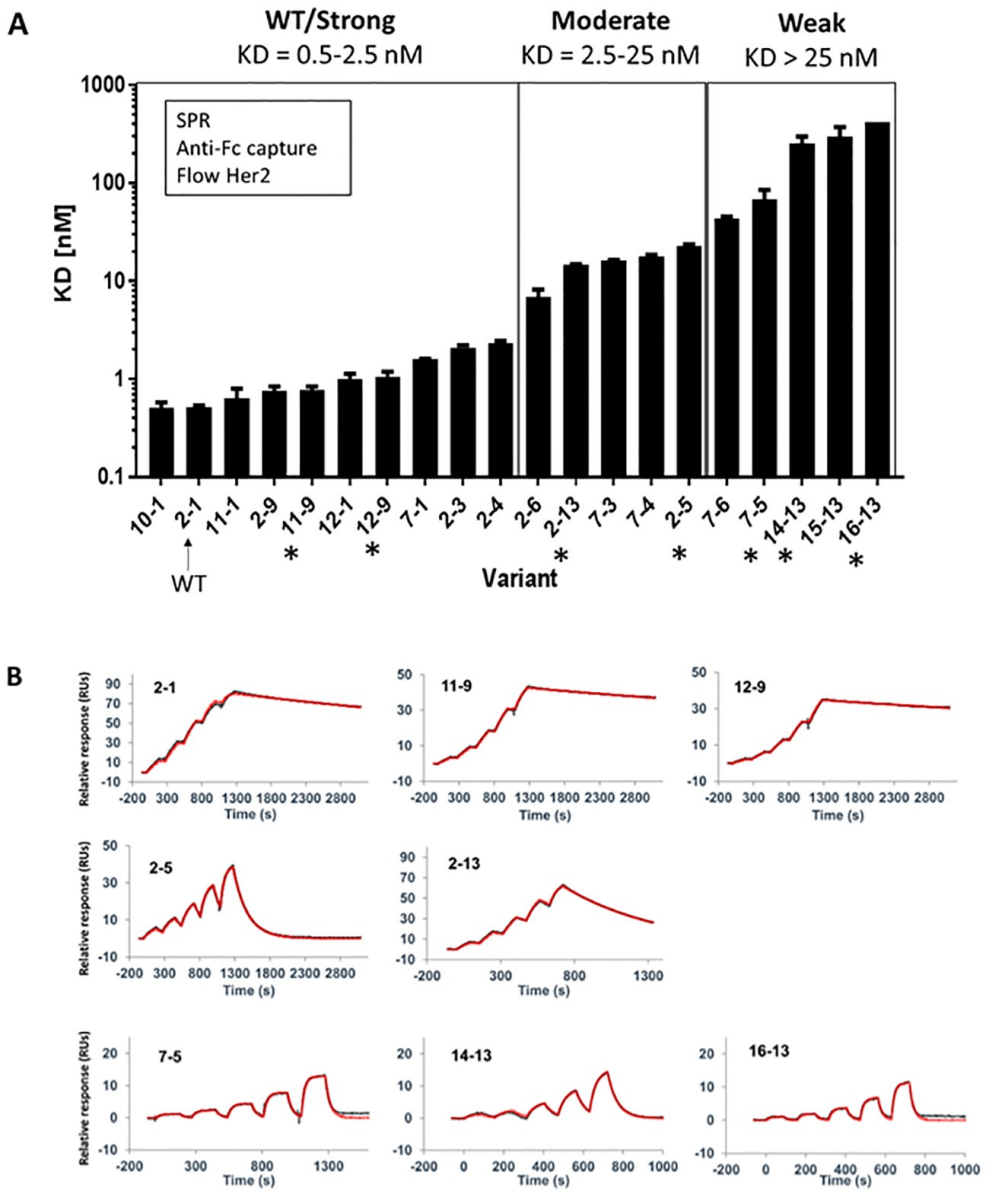
SPR ranking of FSAs and sensorgrams. **(A)** SPR monovalent K_D_ measurements and classification of Herceptin antibody variants. The asterisks indicate variants selected for further analysis. **(B)** Representative sensorgrams from WT/Strong (top), Moderate (middle) and Weak (bottom) variant classifications. The red line indicates the fit modelled to a 1:1 interaction with Her2.

Full-size antibody (FSA) versions of the 20 variants were produced and then analyzed via SPR to determine Her2 binding affinities. Prior to our assays, we also monitored the quality of the FSA samples via size exclusion chromatography (SEC) to ensure purity and rule out biophysical aberrations (i.e. aggregation) that could cause artifacts (S1 Fig). Capturing the antibody on an immobilized anti-Fc antibody surface, followed by flowing soluble Her2 ectodomain in a dilution series, allowed us to measure monovalent Her2 binding. Table 2 and Fig 2A show the SPR-based affinity *K*_D_ values and the ranking of variants from lowest to highest *K*_D_. The variants were classified into three main groups; WT/strong (*K*_D_ = 0.5–2.5 nM), moderate (*K*_D_ = 2.5–25 nM) and weak (*K*_D_ > 25 nM) Her2 binders (Fig 2A). Most variants predicted computationally to be moderate or weak binders (Table 1) fell into their predicted classes when categorized by SPR. The exceptions are 7–1 and 2–3 which were predicted to be moderate but are WT/strong binders, and 7–3 and 7–4 which were predicted to be weak but are moderate binders. Furthermore, the variants predicted to be strong binders (12–9, 11–9, 11–1, 12–1) were not distinguished via SPR from WT-like binders. This may be due to the fact that high affinities in this range may exceed the ability of SPR to distinguish between very slow off-rates. Overall, there was generally a good correlation between the predicted affinity differences, and the affinity differences determined by SPR.

**Table 2:** Antibody variants ranked according to monovalent Her2 binding affinities measured by SPR.

Binding strength	Variant	Mutations	Her2 binding of FSA ^c^	
			*K*_D_ [nM] ^a^	SD ^b^
WT/Strong	10–1	*H*-D98F	0.51	0.07
	**2–1**	**Parent 1 (Herceptin-Fab)**	**0.52**	**0.02**
	11–1	*H*-D98F, *H*-D31K	0.64	0.16
	2–9	*L*-D28K	0.76	0.08
	11–9	*H*-D98F, *H*-D31M, *L*-D28K	0.77	0.07
	12–1	*H*-D98F, *H*-D31M	1.01	0.12
	12–9	*H*-D98F, *H*-D31K, *L*-D28K	1.06	0.13
	7–1	*H*-Y57A	1.60	0.01
	2–3	*L*-F53N	2.10	0.11
	2–4	*L*-Y92A	2.32	0.13
Moderate	2–6	*L*-N30D	6.85	1.36
	**2–13**	**Parent 2 (bH1-Fab)**	**14.74**	**0.17**
	7–3	*H*-Y57A, *L*-F53N	16.12	0.33
	7–4	*H*-Y57A, *L*-Y92A	17.97	0.64
	2–5	*L*-F53N, *L*-Y92A	22.63	1.11
Weak	7–6	*H*-Y57A, *L*-N30D	43.24	2.07
	7–5	*H*-Y57A, *L*-F53N, *L*-Y92A	68.08	16.93
	14–13	*H*-D98W, *H*-G99D	252.75	45.04
	15–13	*H*-G99D	297.75	73.19
	16–13	*H*-G99R	416.70	n = 1

^a^ Average values, and

^b^ standard deviations, from duplicate runs except for variant 16–13.

^c^ Monovalent binding of Her2 ectodomain to FSA variants immobilized on the SPR surface.

Fig 2B and Table 3 show representative Her2 binding sensorgrams and binding kinetic values, respectively, for selected FSA variants from each class. Overall the on-rate for every variant is similar to WT ranging only 5–fold from 6.7 × 10^6^ to 2.7 × 10^5^ M^-1^s^-1^. As mentioned above, the variants predicted to be strong binders (12–9, 11–9) were not distinguished via SPR from WT, possibly due to the fact that these antibodies exhibit very slow off rates (Table 3) that approach the *k*_off_ limit (10^−5^ s^-1^) of the SPR instrument used here (Biacore T200, [17]). Notably, moderate variants 2–5 and 2–13 show faster off-rates and ~30-40-fold higher *K*_D_s, compared to WT. Even faster off-rates are apparent for weak variants 7–5, 14–13 and 16–13 and *K*_D_s between ~130-800-fold higher than WT.

**Table 3:** Her2 binding kinetics of selected antibody variants measured by SPR.

Fab class		Variant	Her2 binding of FSA ^c^			
			*k*_on_ ^a^ [10^5^M^-1^s^-1^]	*k*_off_ ^a^ [10^-4^s^-1^]	*K*_D_ ^a^ [nM]	K_*D*_ SD ^b^ [nM]
*in silico*	SPR					
Strong	WT/Strong	11–9	1.00	0.77	0.77	0.07
Strong	WT/Strong	12–9	0.66	0.70	1.06	0.13
WT	WT/Strong	2–1	2.14	1.12	0.52	0.02
Moderate	Moderate	2–13	1.00	7.41	14.74	0.17
Moderate	Moderate	2–5	2.68	60.80	22.63	1.11
Weak	Weak	7–5	1.60	154.00	68.08	16.93
Weak	Weak	14–13	0.78	96.80	252.75	45.04
Weak	Weak	16–13	1.20	499.00	416.70	n = 1

^a^ Average values, and

^b^ standard deviations, from duplicate runs except for variant 16–13.

^c^ Monovalent binding of Her2 ectodomain to FSA variants immobilized on the SPR surface.

### Flow cytometry binding assessments of Herceptin variants panel in cells with high versus low Her2 density

In the following studies, cell lines having very low (MCF7), low (JIMT-1) and high (SKOV3) Her2 densities were used to further screen the mutant panel and distinguish binding affinities, including avidity effects, using flow cytometry of live cells. Table 4 shows the relative Her2 densities as determined by monovalent binding of Herceptin Fab on these cells, which range from 1,800 to 200,000 receptors per cell. In cell binding assays, we predicted that the lower receptor density of Her2 on MCF7 or JIMT-1 cells should minimize avidity effects and allow assessment of monovalent binding, whereas the higher Her2 density of SKOV3 cells may reveal avidity effects as evidenced by relative apparent binding affinities (*K*_D_) and maximal binding levels (saturation binding levels, *B*_max_ = maximum Median Fluorescence Intensity (MFI)). Jurkat cells are virtually Her2 negative and provide a good negative control to evaluate the Her2 specificity.

**Table 4:** Relative Her2 receptor levels on cells determined via flow cytometry.

Cells	MFI ^a^	% Her2^b^	Molecules/cell ^c^
SKOV3	7280	100.0	200,000
JIMT1	400	5.5	11,000
MCF7	68	0.9	1,800
Jurkat	13	0.2	400

^a^ Mean fluorescence index determined by cell binding of WT-Herceptin-Fab + AlexaFluor488-(Fab)^2^ secondary.

^b^Her2 percentage is based on assumed 1:1 binding of Herceptin Fab. On high density Her2 cells (e.g. SKOV3) this may be underestimated due to crosslinking of Fabs by AlexaFluor488-(Fab)^2^.

^c^ Value based on calculated number of molecules in SKOV3 cells (derived from Ref. [18]).

Table 5 and S2 Fig **panels A-D** show the apparent binding *K*_D_s and maximum binding levels (*B*_max_) for the antibody variant classes in the low-Her2 density MCF7 and high-Her2 density SKOV3 cells, as determined by flow cytometry. For ease of comparison, the order of variants in Table 5- corresponds to the SPR ranking order shown above in Fig 2A. Four of the 5 weak variants (7–5, 14–13, 15–13 and 16–13) exhibited poor binding in MCF7 cells with their *K*_D_s only being approximated (≥ 200 nM). In MCF7 cells, the apparent cell-binding *K*_D_s for the moderate Her2 binders were significantly higher (range = 0.47–0.87 nM) compared to WT/strong binders (*K*_D_ range = 0.02–0.52 nM). Notably, variants 11–9 and 12–9, computationally predicted as the strongest Her2 binders, showed the lowest apparent *K*_D_s (0.02 and 0.04 nM, respectively) compared to the other binding variants. Overall, scanning the classes of Her2 binding variants from WT/strong to weak (left to right, S2 Fig **panel A**), the progressive trend is an increase in *K*_D_.

**Table 5:** Binding parameters *K*_D_ and *B*_max_ determined from flow cytometry.

FSA class (based on SPR & flow cytometry)	Variant	MCF7 cells		SKOV3 cells	
		*B*_max_ [MFI]	*K*_D_ [nM]	*B*_max_ [MFI]	*K*_D_ [nM]
WT-similar	10–1	697	0.28	26698	1.26
WT	2–1	668	0.36	27418	1.67
WT-similar	11–1	710	0.20	26888	1.02
WT-similar	2–9	746	0.43	26946	1.29
Strong	11–9	700	0.02	28223	0.80
WT-similar	12–1	644	0.19	27497	0.98
Strong	12–9	763	0.04	32350	0.90
WT-similar	7–1	673	0.52	27121	2.27
WT-similar	2–3	636	0.30	27790	1.95
WT-similar	2–4	656	0.39	27379	1.98
Moderate	2–6	588	0.51	26948	2.58
Moderate	2–13	644	0.87	26549	2.41
Moderate	7–3	527	0.67	26546	2.34
Moderate	7–4	539	0.47	27027	2.41
Moderate	2–5	532	0.69	26561	1.98
Weak	7–6	503	0.95	26361	3.16
Weak	7–5	455	594 ^a^	21903	3.50
Weak	14–13	NR	200 ^a^	21950	2.76
Weak	15–13	260 ^a^	293 ^a^	20420	6.42
Weak	16–13	NR	2075 ^a^	18072 ^a^	289 ^a^

^a^ Approximated value.

NR: not reached.

In the high-Her2 SKOV3 cells, WT Herceptin (variant 2–1) exhibited a slightly weaker binding affinity relative to that observed in MCF7 cells (1.7 nM versus 0.4 nM) (Table 5). Importantly, the binding of weak variants 7–5, 14–13 and 15–13 on SKOV3 cells significantly improved compared to MCF7 cells, with *K*_D_s ranging between 2.8–6.4 nM (versus ≥ 200 nM on MCF7 cells). The weakest variant, 16–13, also bound better to SKOV3 cells compared to MCF7 cells. This suggests that, on high Her2 density SKOV3 cells, avidity promotes cell surface binding of the weak antibodies.

Based on *in silico* predictions and the SPR and flow cytometry screens, the following subpanel was selected as representative of the different binding classes: strong (12–9, 11–9), moderate (2–5, 2–13) and weak (14–13, 7–5, 16–13). These candidates were further analyzed in competitive cell-binding, internalization, and ADC assays, and were benchmarked against WT Herceptin (2–1).

### Cell-binding behavior of selected candidates

**Fig 3:**
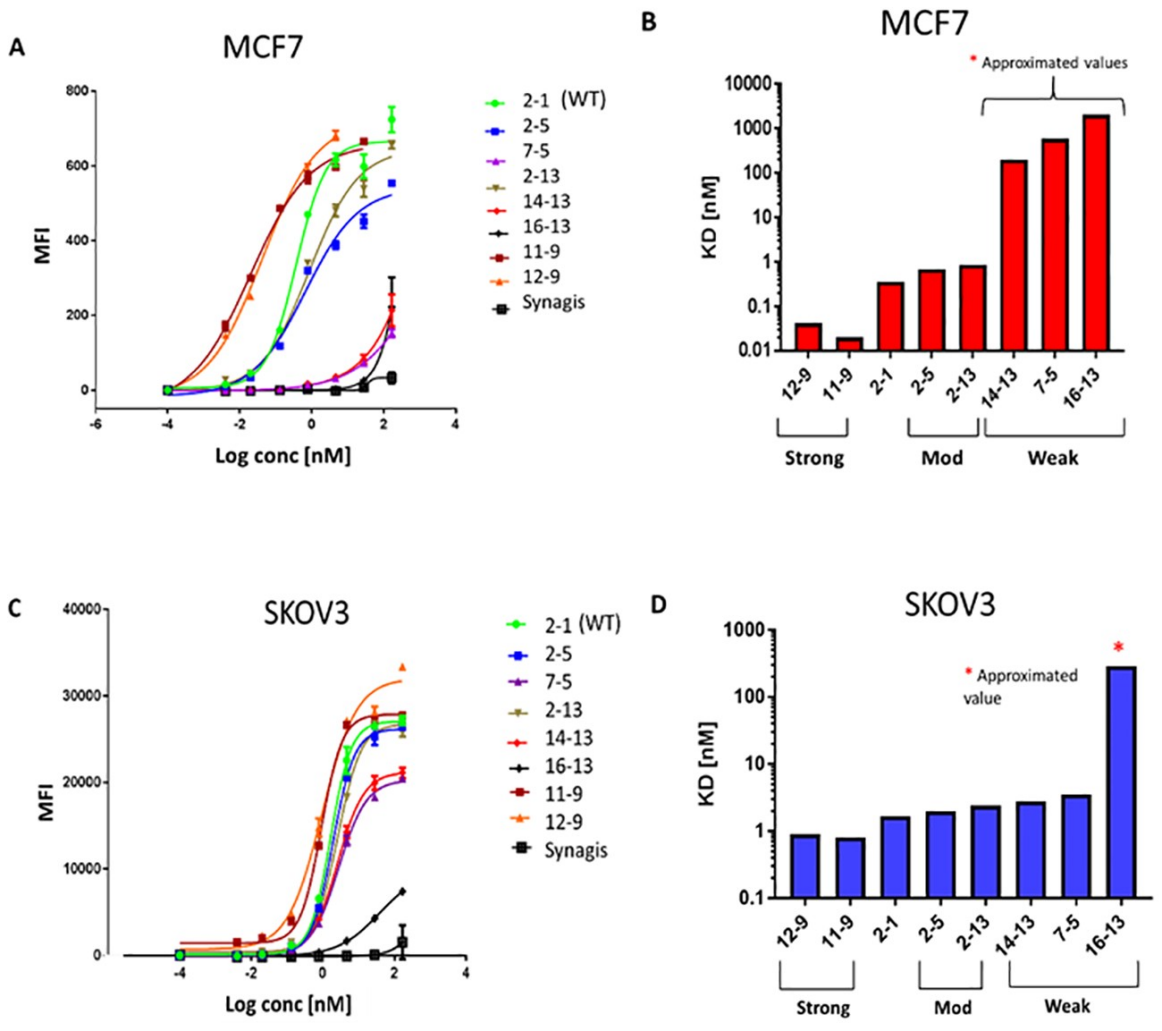
Saturation binding curves and apparent binding affinities (K_D_s) of selected antibody variants in low-Her2 (MCF7) and high-Her2 (SKOV3) cells, as determined via flow cytometry. **(A, B)** MCF7 cells. **(C, D)** SKOV3 cells.

Fig 3A and 3C show binding curves for the 8 selected antibodies in low-Her2 MCF7 and high-Her2 SKOV3 cells, respectively, as determined by flow cytometry. Synagis antibody (aka Palivizumab), which is directed against an antigen encoded by respiratory syncytial virus (RSV), was included as an IgG1 isotype, negative control to assess non-specific binding. For Her2 binders 11–9 and 12–9, the last 1 or 2 points were above the WT binding plateau in MCF7 cells (>1 nM antibody concentration), likely due to some non-specific binding on this cell line at the high concentrations, and were excluded from the generated curves. The curves were used to determine the binding affinity *K*_D_s and *B*_max_ values reported in Table 5. The binding classes and *K*_D_ values in MCF7 cells are summarized in Fig 3B. Together, Fig 3A and 3B illustrate the clear distinction between the different classes of Her2 binders on MCF7 cells. Strong variants 11–9 and 12–9 exhibit similar binding affinities (*K*_D_ = 0.02 and 0.04 nM, respectively) while moderate variants 2–5 and 2–13 have affinities approximately one order of magnitude lower and are similar to WT (*K*_D_ range ~ 0.3–0.9 nM). The weak variants bind poorly (*K*_D_s are greater than 200 nM and cannot be determined accurately for the concentration range of antibody used in the study).

In contrast to MCF7 cells, and as expected for higher avidity being exhibited by SKOV3 cells, the *K*_D_s for most of the variants (from strong to weak) fall into a similar range of apparent affinity (*K*_D_ ~ 1–6 nM) (Fig 3C). Noticeably, only the weakest variant 16–13 stands out from the others, with a *K*_D_ greater than 200 nM (Table 5). In SKOV3 cells, there is an apparent decrease in the *B*_max_ levels for the weaker variants compared to WT and strong variants (Fig 3C and S3 Fig **panel B**), suggesting differences in their engagement of Her2 receptors at the cell surface (see the Discussion section).

In the cell binding assays presented so far, it is possible that, in the case of weak binders with fast off-rates, the washing procedure may have unintentionally removed some of the bound antibody. In order to measure relative binding strengths of the mutants in a manner that is less sensitive to fast off-rates, we performed competitive binding. This assay measures the ability of variant antibodies to compete with fluorescently-labeled WT antibody for binding to SKOV3 cells. Increasing concentrations of variant antibody were combined with a constant subsaturating amount of fluorescently-labelled WT. The sample mixture was then added to SKOV3 cells, incubated a 4°C to allow surface binding and cells were subsequently analyzed by flow cytometry to measure the effect on surface-bound fluorescence signal. S4 Fig shows the competition binding curves and respective IC_50_s for each variant antibody, as compared to WT (homologous binding) and Synagis controls. Strong binders 12–9 and 11–9 both competed more strongly for binding than the WT control. Moderate binders 2–5 and 2–13 competed less strongly than the WT control. Weak binders 7–5 and 14–3 competed even less strongly, with 16–13 appearing as the weakest. The competition IC_50_s reveal a clear discrimination amongst the Her2 binding antibody classes in accord with the increased competitive binding strengths of the variant antibodies. Together these results confirm the ranking order of the chosen candidate variants and designated classifications. In addition, the ability of these antibodies to compete with WT Herceptin (2–1) for binding to Her2 on SKOV3 cells verifies that their mutated residues did not alter specificity for their shared Her2 epitope.

### Internalization screens of candidate antibodies using high content imaging

To assess the contribution of Her2 binding affinity on cell internalization, endocytosed antibody was detected using anti-human IgG secondary antibody labelled with pH-sensitive dye pHAb and quantified using high-content imaging methodology. This dye emits a strong fluorescence signal in the low pH environment in endosomes and lysosomes but not at neutral pH (pH ~7) exterior to the cell. In this indirect assay, increasing concentrations of antibody were combined with pHAb secondary antibody and then the mixture was added to high-Her2 SKOV3 cells in 96-well plate format. The cells were incubated at 37°C for 18 h to allow for internalization, followed by detection of fluorescence using an ImageXpress imager. This method indirectly measures the total intracellular accumulation of internalized antibody over time.

**Fig 4:**
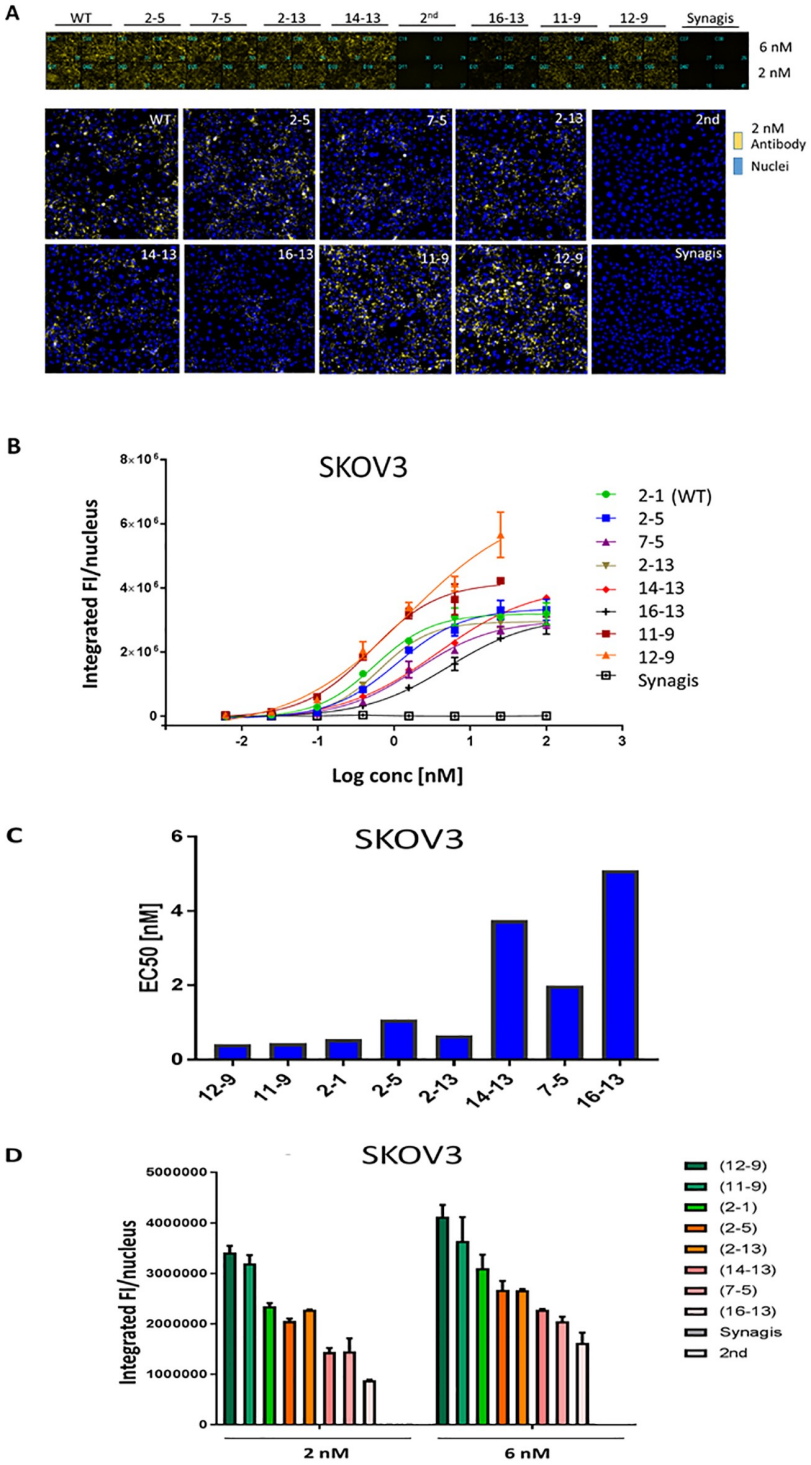
Internalization of antibody variants in SKOV3 cells as measured using indirect detection by pHAb-secondary antibody and high content imaging. **(A)** Well-view images of SKOV3 cells treated with the indicated variant antibody plus pHAb-labelled secondary antibody at 37°C for 18 h (top panel). 2^nd^ = secondary antibody alone, Synagis = IgG1 control. The bottom panel shows representative processed images (yellow = antibody; blue = nuclei). **(B)** Internalization dose response curves, plotting integrated fluorescence intensity/nucleus versus Log_10_ concentration of antibody. **(C)** Internalization EC_50_s (concentration at which the amount of internalized antibody is 50% relative to maximum accumulation) of variants derived from dose response curves. **(D)** Intracellular fluorescence intensities at selected concentrations of antibody (2 and 6 nM).

The top of Fig 4A shows the pHAb fluorescence signals in SKOV3 cells treated with the different antibody variants. Shown are duplicate well-view images of cells at selected doses (2 and 6 nM) from the concentration series. The bottom of Fig 4A shows representative, processed images showing antibody fluorescence (yellow) and cell nuclei (blue, Hoechst stain). Imaging software identifies and quantifies fluorescence signals from small punctate objects, which represents internalized antibody. This is measured as a total integrated Fluorescence Intensity which is normalized to the nuclear count (FI/nucleus). Fig 4B shows dose-dependent, internalization response curves for the mutants in SKOV3 cells. Internalization efficiencies derived from these curves (measured as EC_50_, concentration at which the amount of internalized antibody is 50% relative to the maximum), are summarized in Fig 4C and S1 Table.

The internalization curves for strong (12–9, 11–9) and moderate variants (2–5, 2–13) appear to be distinct from each other (Fig 4B). Noticeably, internalization levels are higher at every dose for strong Her2 binders, compared to the moderate and WT antibodies. Nevertheless, internalization EC_50_s of strong and moderate variants are similar to WT (EC_50_ range = 0.41–1.08 nM, Fig 4C). In contrast, internalization of the weak variants (14–13, 7–5, 16–13) is less efficient (EC_50_ range ~2–5 nM) although maximum accumulated levels after 18 h are similar compared to moderate and WT Her2 binders (~4 × 10^6^ integrated FI/nucleus, Fig 4B). Fig 4D compares the fluorescence levels between the different variants at 2 selected doses (2 and 6 nM) that are typically below the target-independent toxicity IC_50_s normally observed for irrelevant ADCs in an ADC cytotoxicity assay (e.g. Synagis-DM1 IC_50_ ~ 40 nM). At these efficacious doses, the level of internalized antibody correlates directly with binding strength of the mutants.

To validate our indirect internalization screening results, which used a pHAb-labelled secondary antibody, we next performed direct internalization in SKOV3 cells using antibody variants directly conjugated to pHAb. Following conjugation, the dye/antibody ratios (DARs) were similar between the variants (~1.4–2.3 dye molecules/antibody) and sample aggregation was checked via SEC (> 93% monomer content, S2 Table). It should be noted that image acquisition parameters were adjusted according to the lower background signal and a wider dynamic range was achieved in this direct assay, compared to the indirect assay.

**Fig 5:**
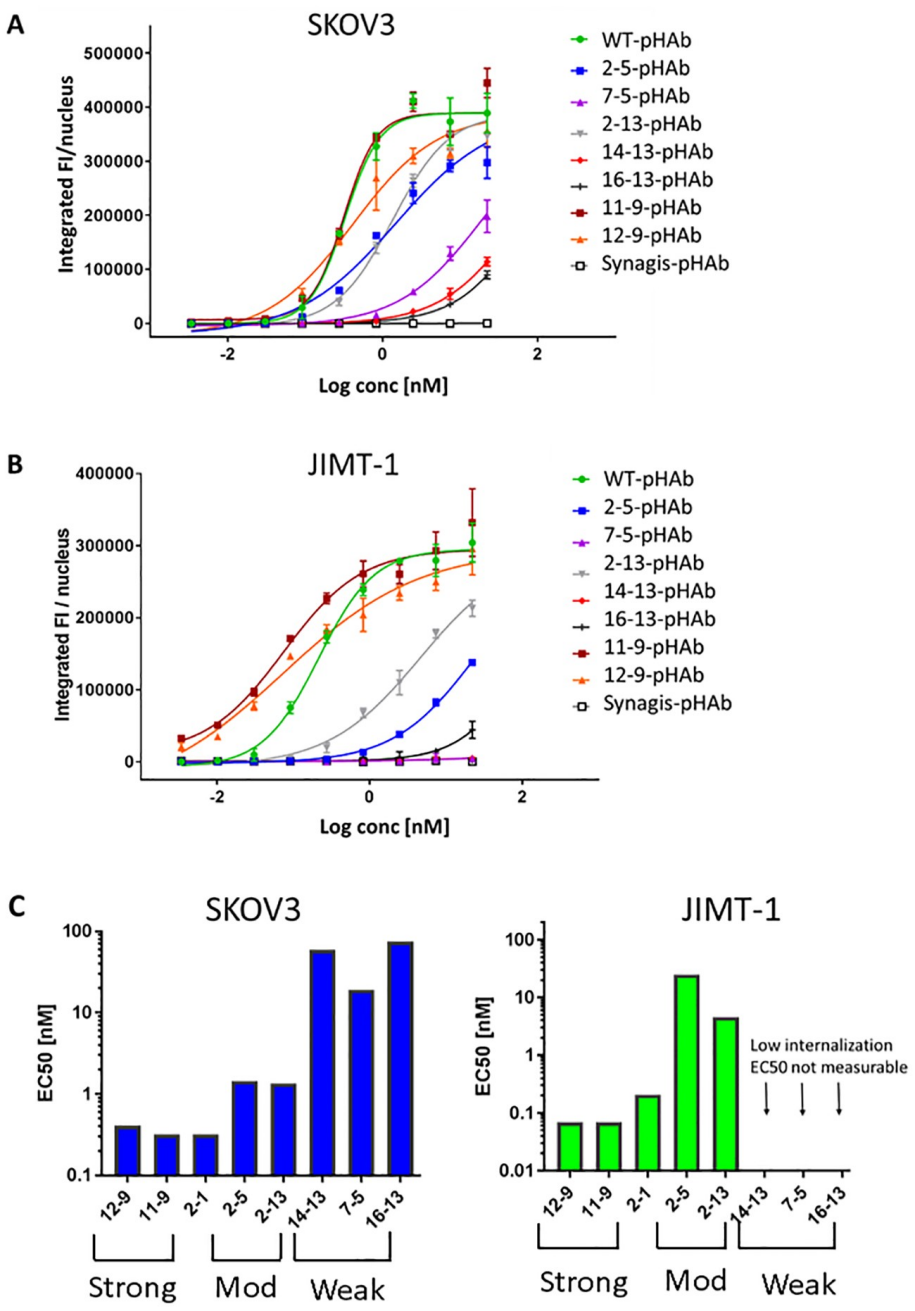
Internalization in SKOV3 and JIMT-1 cells as measured by direct detection of pHAb-labelled antibody variants and high content imaging. Internalization dose response curves of variants in **(A)** SKOV3 cells and **(B)** JIMT-1 cells, following treatment with antibody at 37°C for 18 h. **(C)** Internalization EC_50_s of variants.

Fig 5A shows DAR-normalized, internalization curves for pHAb-antibody variants in SKOV3 cells treated at 37°C for 18 h. Fig 5C, left panel, and S1 Table summarize the EC_50_s derived from these curves. The wider dynamic range allowed us to distinguish between the EC_50_s of strong/WT versus moderate and weak Her2-binding antibody classes more readily than observed in the indirect internalization screen (compare direct, Fig 5C, with indirect, Fig 4C).

We next attempted to assess Her2 avidity effects on internalization of antibody variants by comparing SKOV3 versus MCF7 cells. However, in both direct and indirect internalization assays, fluorescence signals for moderate and weak mutants were not sufficiently above background in MCF7 cells, likely due to their low Her2/binding levels (S5 Fig). We decided to use JIMT-1 cells, which have Her2 levels that are low, but are ~6-fold higher than in MCF7 cells (Table 4). In contrast to SKOV3 cells, in JIMT-1 cells the direct internalization curves for moderate variants 2–5 and 2–13 were well separated from strong variants and WT (Fig 5B), i.e. internalization efficiencies of the moderate Her2 binding variants were markedly lower than strong variants and WT (by ~ 60-370-fold, Fig 5C, right panel, S1 Table). Internalization for the weak binding variants in JIMT-1 cells was very low and not measurable.

Together, our binding and internalization results indicate that high Her2 density and avidity at the cell surface greatly improves Her2 binding and internalization of moderate variants to levels approaching that of WT Herceptin. Weak variants also bind better when Her2 density/avidity is high and this promotes internalization, although the levels of internalized antibodies are less than for WT and moderate variants. Strong mutants always bind well and internalize efficiently, independently of avidity.

### Drug-conjugate screens of antibody variants in cells with different Her2 densities

The antibody variants were next evaluated for ADC potential using an indirect ADC cytotoxicity assay, comparing activities in SKOV3, JIMT-1 and MCF7 cells and using two different drugs; DM1 and MMAE. MCF7 cells were used as a surrogate for ‘normal’ low-Her2 tissue cells and an indicator of potential ADC toxicity [19]. We compared MCF7 and JIMT-1 cells in order to explore possible thresholds of Her2-mediated avidity. We wished to determine whether the slightly higher Her2 levels on JIMT-1 cells might be sufficient to promote ADC activity of the weaker variants, as suggested above in our internalization data with JIMT-1 cells.

Control Her2-negative Jurkat cells were first tested to assess for Her2-specific versus non-specific ADC effects. In the indirect ADC assay, increasing concentrations of antibody variant complexed with DM1-conjugated secondary antibody was added to cells in a 384-well plate format, followed by incubation at 37°C for 5 days. DM1 drug alone was also tested as a positive control. Cell viability was measured via ATP detection using Cell Titer-Glo as a readout. In Jurkat cells, no cytotoxicity was detected up to 10 nM for any of the antibody variants, indicating that the ADC responses reported below in SKOV3, JIMT-1 and MCF7 cells are indeed Her2 specific (S6 Fig). Only the active DM1 drug control resulted in significant cytotoxicity in the sub-nM range.

S7A–S7C Fig shows growth inhibition curves from the secondary-DM1 ADC screening assay for antibody-treated SKOV3, JIMT-1 and MCF7 cells, plotted as % surviving fraction (relative to non-treated cells) versus antibody concentration. The DM1 ADC IC_50_s derived from these curves are summarized in S8 Fig **panels A-C** and Table 6. In SKOV3 cells, the moderate and weak variants all show lower potencies (IC_50_ range = 0.022–0.106 nM) compared to either WT Herceptin (2–1; IC_50_ = 0.004 nM) or the direct DM1-conjugated Herceptin control (T-DM1; IC_50_ = 0.003 nM). In contrast, the strong variants are highly potent (IC_50_ range = 0.0001–0.0004 nM), exhibiting IC_50_s approximately one order of magnitude lower than WT. The ADC potencies for all the variants are well above background, as determined by the isotype antibody control (Synagis; IC_50_ = 4.5 nM).

**Table 6:** Potencies of antibody variants indirectly conjugated to the DM1 and MMAE cytotoxic drugs.

FSA class	Variant	DM1						MMAE			
		SKOV3		JIMT1		MCF7		SKOV3		JIMT1	MCF7
		IC_50_ [nM]	Fold potency ^a^	IC_50_ [nM]	Fold potency ^a^	IC_50_ [nM]	Fold potency ^a^	IC_50_ [nM]	Fold potency ^a^	IC_50_ [nM]	IC_50_ [nM]
Strong	12–9	0.0004	12200	1.3	14	3.1	4	0.014	7475	0.1	0.5
Strong	11–9	0.0001	37500	6.7	3	3.0	5	0.008	13149	0.2	0.4
WT	2–1	0.0040	1011	6.2	3	7.8	2	0.056	1855	27.0	48.3
Moderate	2–5	0.0220	201	8.7	2	8.1	2	0.107	965	24.4	20.6
Moderate	2–13	0.0210	215	7.4	3	8.2	2	0.078	1322	10.3	35.6
Weak	14–13	0.0250	182	16.2	1	22.1	1	0.095	1092	11.6	3.4
Weak	7–5	0.0340	132	15.7	1	5.6	2	0.204	509	10956.0	32.4
Weak	16–13	0.1060	42	16.3	1	14.6	1	0.173	598	42.6	84.1
	T-DM1	0.0030	1668	4.9	4	17.5	1	ND	ND	ND	ND
	Synagis	4.5040	1	18.7	1	13.9	1	103.600	1	0	0

^a^ Fold potency = IC_50_ Synagis / IC_50_ Variant.

ND: not determined.

In the DM1 ADC screen with JIMT-1 and MCF7 cells, the strong variants again exhibited the highest potencies (low nM), that were ~2-5-fold more potent than WT and moderate antibodies. Nevertheless, the DM1 potencies for WT and moderate variants were slightly (2-3-fold) stronger than the irrelevant antibody control (Synagis) background in these low-Her2 cells (Table 6, S8B and S8C Fig). In contrast, in most cases the DM1 potencies for the weak antibodies were closer or identical to that seen with the irrelevant antibody control (Synagis) background in these low-Her2 cells.

A similar relative ranking of the antibody variants is observed between the DM1 and MMAE screens in the high-Her2 SKOV3 cells (compare DM1 and MMAE, S8 Fig **panels A** and **D**, respectively). Similar to DM1, with MMAE the strong variants showed the highest potencies in SKOV3 cells (MMAE IC_50_s ~ 0.01 nM) and moderate and weak variants also showed significant potencies (MMAE IC_50_ range ~ 0.1–0.2 nM). In the MMAE screen with JIMT-1 and MCF7 cells the moderate and weak (and WT) antibodies all showed low potencies (majority > 10 nM, S8E and S8F Fig, Table 6). These results indicate that with MMAE the WT antibody and all the weaker Her2 binding variants showed only low, background toxicities in JIMT-1 and MCF7 cells, hence they are not likely to exibit target-dependent toxicity with this drug in low-Her2 tissues.

**Fig 6:**
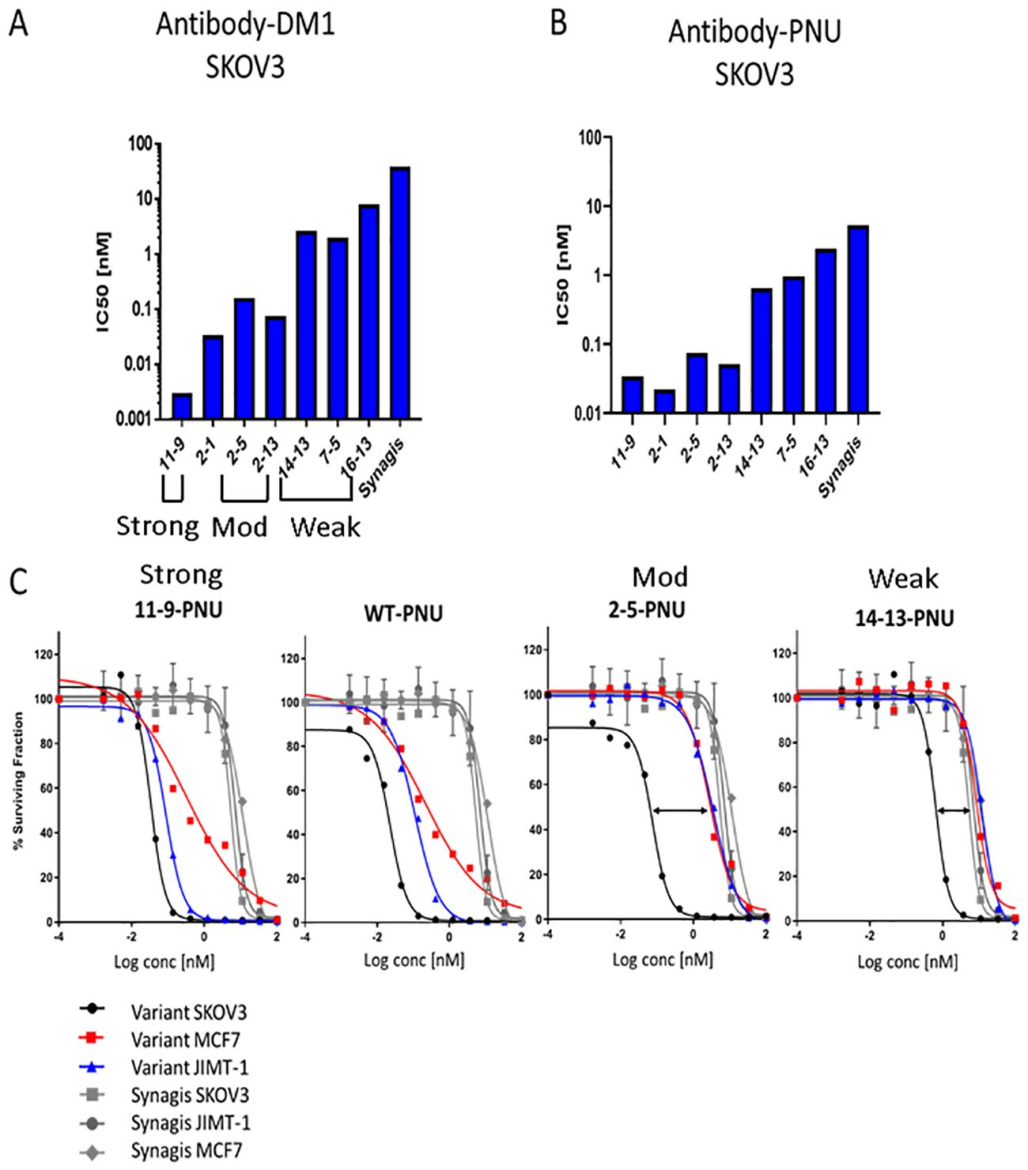
Direct ADC cytotoxicity of DM1 and PNU conjugated variants in Her2 cells. IC_50_s of DM1-antibodies **(A)** and PNU-antibodies **(B)** in SKOV3 cells. **(C)** Survival curves in SKOV3, JIMT-1 and MCF7 cells treated with the indicated antibody-PNU variant or Synagis-PNU. Double-head black arrows denote the therapeutic window (concentration range between high-Her2 SKOV3 and low-Her2 MCF7 cells). The data are normalized, based on DAR (Drug Antibody Ratio), to account for small DAR differences between antibody variants.

In order to validate the ADC activities of the variants seen in our ADC screens and the advantage of Her2 avidity-mediated selectivity for SKOV3 cells, we performed ADC assays using antibodies directly conjugated to two different drugs and linkers; SMCC-DM1 and the highly potent vc-PNU159682 (PNU). It should be noted that SPR-based assessments of the drug-conjugated antibodies (S3 Table) indicated that Her2 binding of variant 12-9-DM1 was significantly impaired, likely due to amine/drug-coupling to one or both of the two substituted lysines within the binding site of this particular mutant (Table 1). Consequently, 12–9 was not included in our direct ADC assays. The drug-conjugated antibodies were tested for their relative cytotoxic potencies in SKOV3 cells versus JIMT-1 and MCF7 cells (Fig 6A–6C, Table 7, S9 Fig). In the direct ADC assay with DM1-conjugated variants in SKOV3 cells, the variants differed as predicted from the above binding, internalization and indirect ADC assays, having IC50s that ranged from 0.003 to ~ 8 nM (Fig 6A, Table 7, S9 Fig **panel A**). However, in the low-Her2 JIMT-1 and MCF7 cells, the potencies of the DM1-conjugated variants could not be distinguished from the control Synagis-DM1 (S9 Fig **panels B** and **C**), perhaps due to over-riding, non-target mediated ADC uptake by pinocytosis. We proposed that we would see higher on-target potency in the low-Her2 cells if the variants were coupled to a stronger payload, such as PNU, and this would enable separation between the specific versus non–specific effects.

**Table 7:** Potencies of antibody variants directly conjugated to the DM1 and PNU cytotoxic drugs.

FSA class	Variant	DM1		PNU					
		SKOV3		SKOV3			JIMT1		MCF7
		IC_50_ [nM]	Fold potency ^a^	IC_50_^b^ [nM]	Fold potency ^a^	IC_50_^b^ [nM]	Fold potency ^a^	IC_50_^b^ [nM]	Fold potency ^a^
Strong	11–9	0.003	12610	0.041	123	0.08	102	1.24	8
WT	2–1	0.034	1117	0.022	229	0.13	63	0.56	17
Moderate	2–5	0.161	239	0.138	37	3.38	2	4.72	2
Moderate	2–13	0.075	512	0.077	66	0.50	16	2.02	5
Weak	14–13	2.658	14	1.525	3	9.66	1	12.75	1
Weak	7–5	2.007	19	2.078	2	11.80	1	13.66	1
Weak	16–13	8.033	5	3.993	1	9.29	1	15.05	1
	Synagis	38.360	1	5.038	1	8.08	1	9.61	1

^a^ Fold potency = IC_50_ Synagis / IC_50_ Variant

^b^Mean IC50 for n = 3 experiments using PNU conjugated variants

Fig 6, **panels A** and **B** show the IC_50_s for the DM1 and PNU conjugated antibodies, respectively, in SKOV3 cells. The rank orders of the DM1 and PNU antibodies, based on potency, were similar and coincided with that observed in the indirect conjugated-DM1 ADC screen using SKOV3 cells. As previously seen in the internalization assays, the relative potencies between the different variant classes were more clearly separated for the directly conjugated antibodies compared to the ADC screen using a drug-conjugated secondary antibody (compare direct, Fig 6, **panels A** and **B**, with indirect, S7 Fig **panel A**). This is likely due to the additional avidity effect caused by the secondary antibodies and this appears more pronounced on weaker variants than for WT, moderate or stronger Her2 binders (compare fold-potency effects for direct versus secondary DM1-labelled antibodies in SKOV3 cells, Tables 7 and 6, **respectively**).

In SKOV3 cells, ADC variant 11–9 was very potent, having IC_50_s either ~ 10-fold lower or similar to WT (IC_50_ = 0.003 and 0.041 nM when conjugated to DM1 and PNU, respectively, Table 7). However, PNU-conjugated 11–9 and WT both showed significant potencies (low to sub-nM) in MCF7 and JIMT-1 cells, indicating they are likely to be toxic in low-Her2 normal tissues (Table 7). Moderate (2–5, 2–13) and weak (14–13, 7–5) variants also had significant PNU potencies (low to sub-nM) in SKOV3 cells. In contrast to WT and the strong variant, their potencies were low in MCF7 and JIMT-1 cells and ranged either ~2-16-fold above (2–5, 2–13, moderate class) or similar (14–13, 7–5, weak class) to the Synagis background range (~8–10 nM) in these cells, indicating a low toxicity for these variants in low-Her2 cells. The weakest antibody, 16–13, showed the lowest potencies, close to background with both DM1 and PNU in SKOV3 cells.

Fig 6C shows survival curves for the three different cell lines for PNU-conjugated WT and representative variants from each class, compared to Synagis-PNU. The different variant classes were readily distinguished based on the pattern of their potencies in the high versus low Her2 cell lines. Specifically, it can be seen that WT exhibited similar potencies in MCF7 (blue), JIMT-1 cells (red) and SKOV3 cells (black circle). A similar cytotoxicity profile for the three cell lines can also be seen for the strong affinity 11-9-PNU ADC. In contrast, the potencies observed for moderate 2-5-PNU and weak 14-13-PNU ADCs in MCF7 and JIMT-1 cells are close or identical with Synagis-PNU IC_50_s (shown in grey). The right-shift towards non-specific toxicity is most pronounced for weaker variants 14–13 and 7–5, however this is at the expense of lower potency in SKOV3 cells (weak variant IC_50_ range ~ 1.5–2.1 nM) compared to moderate variants 2–5 and 2–13 (IC_50_ range ~ 0.08–0.14 nM) (see SKOV3 PNU values, Table 7). Together, these results illustrate a wider therapeutic window for the moderate antibody variants when conjugated to PNU, as compared to weak variants, but at the expense of some toxicity in the low-Her2 cells above background levels (Fig 6C). Notably, variant 16-13-PNU showed no significant toxicity above Synagis-PNU background in all cell lines examined.

In summary, our results indicate that the antibody variants fall into 3 classes based on affinity and avidity driven effects on binding, internalization and ADC activity: 1) Strong Her2-binding antibodies (12–9 and 11–9) are highly potent ADCs but have the potential to exhibit toxicity in normal cells/tissues, based on their high-binding and toxicity in low-Her2 cells. 2) The moderate and weak classes of antibodies are potent ADCs in high-Her2 cancer cells (e.g. SKOV3) with less potential to be toxic in normal tissues, based on their low ADC potencies in low-Her2 cells (≤ 11,000 Her2/cell). This selective activity on high versus low Her2 cells results from low affinity variants being dependent on Her2 avidity. 3) A further distinction between the relative potencies of moderate (2–5, 2–13) and weak (14–13, 7–5) antibodies was seen when conjugated to payloads DM1 and PNU. With PNU, antibodies 14–13 and 7–5 showed the lowest toxicity on low-Her2 cells, having IC_50_s similar to isotype control antibody.

## Discussion

This study describes a comprehensive regimen for pharmacological characterization and optimal selection of ADC candidates based on their intrinsic binding properties. Selection is based on quantitative measurements of cell-surface binding, internalization efficiency and ADC cytotoxicity. Our test panel was comprised of structure-based computationally-designed Herceptin variants that bind to the same Her2 epitope with different affinities. This evaluation focused particularly on the contributions of varied affinity, in the context of an identical antibody scaffold, and varied cell-surface Her2 density/avidity.

Despite their high activity in cell line models, preclinical studies using naked antibodies have shown that antibodies with very strong affinity for their respective target can exhibit poor tumor penetration, hence low therapeutic efficacy against solid tumors *in vivo*. This is attributed to strong binding to target antigen on the cells at the tumor periphery, thus preventing further diffusion of the antibody into the tumor mass, a phenomenon termed the “binding-site barrier” [20, 21]. Furthermore, it was noted in the case of anti-Her2 antibodies, that after binding target antigen in the peripheral tumor cells, strong-affinity antibodies promoted enhanced internalization and degradation [13]. When purposing an antibody as an ADC, such routing towards degradation and drug release is preferred. Hence, we deemed it important to consider both strong and weak affinity Herceptin antibodies in our evaluation, combined with a careful determination of their relative ADC efficacies and toxicities.

In our criteria for selection of ADCs, we sought antibody candidates that were ‘just right’ in terms of selective binding, intracellular accumulation, and cytotoxicity in high-Her2 SKOV3 cells versus low-Her2 cells (MCF7 cells were chosen as a surrogate for low-Her2 normal cells). Taken together, our results identified two weak Her2-binding antibody candidates (7–5, 14–13) that best fit these criteria, as exemplified for the PNU class of ADCs. Nevertheless, if a less potent drug is used, the moderate variants may be preferred in order to achieve efficient killing of high-Her2 tumors *in vivo*. For example, the moderate variants (2–5, 2–13) conjugated to DM1 showed significant potencies against SKOV3 cells (IC_50_ range ~ 0.08–0.16 nM) (Table 7).

Our cell-binding experiments showed that both moderate (2–5, 2–13) and weak (7–5, 14–13) antibodies exhibited binding in SKOV3 cells that was similar to WT (*K*_D_ range ~1–4 nM, Fig 3D). In contrast, the weak antibodies bound poorly to MCF7 cells (*K*_D_s of weak antibodies were > 100-fold higher than for moderate and WT antibodies, Fig 3B). These results show the significant contribution of Her2 avidity in SKOV3 cells for improving binding of the weak antibodies. The selective binding of the weak antibodies to high-Her2 cells versus weak binding to low-Her2 cells suggests a better safety profile compared to the moderate class and WT.

Paradoxically, the apparent *K*_D_ values determined for WT Herceptin, WT-similar, strong and moderate variants, by cell-binding to SKOV3 cells were somewhat higher than for MCF7 cells (Table 5). This is counterintuitive, based on the respective high versus low Her2 densities on these cells and expected avidity effects. This behavior was previously seen for an anti-EGFR antibody and was attributed to experimental limitations due to an insufficient incubation period for the antibody to achieve equilibrium binding (estimated to require several days) [12]. In addition, we anticipate some contribution of antibody depletion at low antibody doses (i.e. <1 nM), resulting in less available antibody per Her2 receptors on SKOV3 cells compared to MCF7 cells. Despite this limitation, the cell-binding assays were informative as a screening tool for ranking antibodies and identifying Her2 avidity effects.

**Fig 7:**
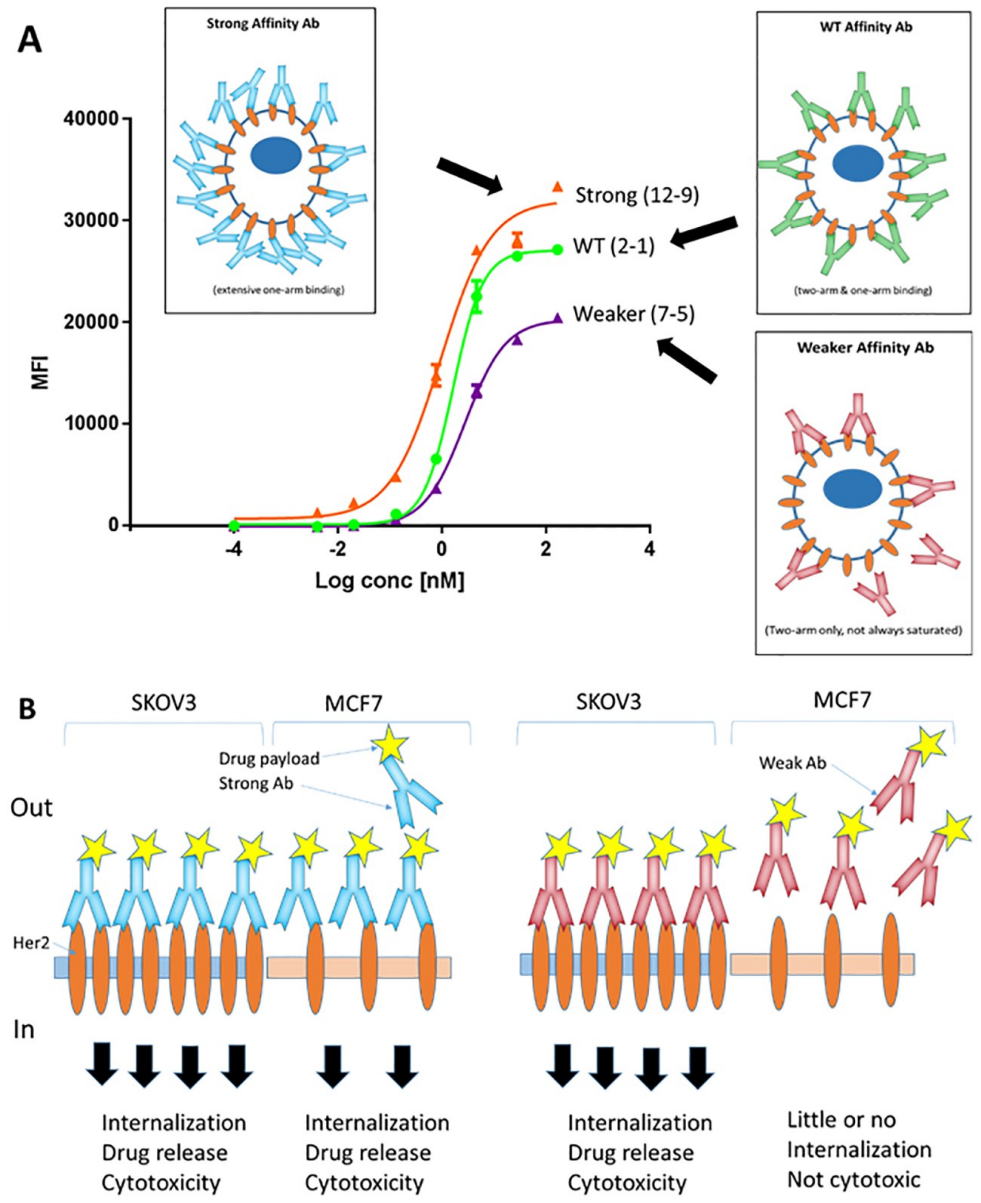
**(A) Saturation binding of affinity-modulated antibodies on high-density Her2 cells**. Shown are binding curves for strong (12–9) and weak (7–5) affinity variants and WT antibody that show the different B_max_ levels attained by these antibodies on SKOV3 cells, as determined via flow cytometry. The schematics (boxes) illustrate how affinity affects the number of antibodies and Her2 receptors (brown oval circles) engaged at the cell surface. **(B) Schematic that illustrates Her2 avidity resulting in selective binding of weak-affinity ADC to Her2 receptors on SKOV3 tumor cells and cytotoxicity**. Left panel: strong-affinity ADC (e.g. variant 11–9 or 12–9) readily binds Her2 receptors on both SKOV3 and MCF7 cells, resulting in cytotoxicity and cell death. Right panel: weak affinity ADC (e.g. variant 7–5 or 14–13) binds well to SKOV3 cells, due to high-density Her2 avidity, but binds poorly to low-density Her2 MCF7 cells, resulting in low cytotoxicity in MCF7 cells.

The maximum binding levels (*B*_max_) attained by the weak antibodies 7–5 and 14–13 were lower than *B*_max_ levels seen for strong, moderate and WT antibodies in SKOV3 cells (Fig 3C). This is likely due to differential receptor engagement by the weak versus stronger binders, as alluded to by Harms et al. [11] based on their cell-binding assays using EGFR-binding antibodies with different affinities [11]. As illustrated in Fig 7A, at high concentrations, bivalent crosslinking of Her2 receptors by an antibody with strong affinity (e.g. 12–9) is presumably competed away by monovalent binding, giving rise to a higher number of antibodies bound at the cell surface. WT (2–1) antibody, being weaker than 12–9, exhibits less monovalent binding and hence a lower number of antibodies bind to the surface (i.e. lower *B*_max_, compared to 12–9). In contrast to strong and WT antibodies, weak antibody (e.g. 7–5) can only bind bivalently, due to fast intrinsic dissociation rate of monomer, resulting in the lowest *B*_max_. Support for high intrinsic binding and receptor occupancy of the strong variants (11–9 & 12–9) and WT was seen in binding experiments with SKOV3 cells using monovalent Fab versions (S10 Fig) in which the strong and WT Fabs reached a similar maximum binding level (presumably full receptor occupancy), with the strong variants exhibiting higher affinity (binding curves shifted left compared to WT). In the absence of avidity, monovalent moderate and weak Fab variants did not attain maximum binding even at the highest concentration used (1 μM). Despite weaker binding compared to stronger antibodies (and WT), it is apparent that the decreased number of Her2 receptors engaged by bivalent 7–5 and 14–13 antibodies on the surface of SKOV3 cells was sufficient for their internalization and growth inhibition in these cells when coupled to potent drugs.

The internalization assays demonstrated that weak antibodies 7–5 and 14–13 internalize moderately well in SKOV3 cells whereas internalization was very low and not measurable in low-Her2 JIMT-1 cells when detected using pHAb fluorescence (Fig 5C). In contrast, the moderate affinity antibodies did internalize in JIMT-1 cells, although with reduced efficacies compared to SKOV3 cells. These results suggest that minimal thresholds of antibody binding and Her2 receptor engagement are required for efficient internalization and ADC activity and that full receptor occupancy on the surface of the cells is not necessary for maximal effect, as elucidated in the “spare receptor” concept in pharmacology [22]. In the case of the strong antibodies (and WT), presumably these thresholds are attained even in the lower Her2 JIMT-1 cells, based on the significant internalization observed for these variants (strong and WT antibody internalization EC_50_s < 1 nM in JIMT-1 cells, S1 Table).

As expected, and based on both the indirect ADC screens and assays using drug-conjugated antibodies, strong antibodies 12–9 and 11–9 exhibited the highest potencies compared to the lower affinity antibody classes in all Her2 cell lines tested (S7 Fig and Fig 6). These strong variants were extremely potent in SKOV3 cells and also showed significant toxicities in the low-Her2 MCF7 cells with all 3 drugs tested; DM1, MMAE and PNU (Tables 6 and 7). These results flag these strong variants as likely to show toxicity as ADCs in normal cells and tissues.

Based on the indirect ADC screens alone, the weak and moderate class of variants showed similarly potent cytotoxicities in SKOV3 cells (DM1 and MMAE IC_50_s ~ sub-nM range) and much lower potencies on low-Her2 cells, indicating their high potential as ADCs (Table 6).

Using directly DM1-conjugated antibodies in SKOV3 cells, the potencies of the moderate and weak classes of antibodies were further distinguished, differing by ~ one log (IC_50_s of moderate (2–5, 2–13) and weak (14–13, 7–5) variants ranged ~ 0.1–0.2 nM and 2–3 nM, respectively, Fig 6A, Table 7). These results illustrate that ADC screens using secondary antibodies conjugated to drugs such as DM1 and MMAE are useful as a first-round tool to identify ADC hits. Subsequently, direct drug conjugation is required to resolve ADC potency differences between lower affinity candidates (in this case *K*_D_s ranging ~ 20–250 nM).

It should be noted that, for antibodies conjugated to DM1 or MMAE, it is important to evaluate potencies attributed to the different linker-drug combinations. For example, DM1 conjugated 2–1 (WT) and 2–13 (moderate) antibodies showed high potencies that were distinguishable in SKOV3 cells (IC_50_s = 0.03 and 0.08 nM, respectively) and were well above Synagis-DM1 background (IC_50_ = 38 nM) (Table 7). In contrast, the potencies of MMAE conjugated 2–1 and 2–13 antibodies were similarly low in SKOV3 cells (IC_50_s ~ 100 nM, S11 Fig) and close to Synagis-MMAE background.

Overall, our results indicate that Herceptin antibody variants with moderate and weak affinity for Her2 are good ADC candidates with potential advantage over strong antibodies. In particular, weak variants 7–5 and 14–13 are identified as the most promising PNU ADC candidates for treating high Her2 cancers based on the following criteria: 1) they show selective binding to SKOV3 cells and significant (sub nM) ADC potencies and 2) they exhibit the lowest toxicities in low-Her2 cells compared to strong, moderate and WT Herceptin. Fig 7B summarizes the concept of exploiting Her2 avidity to achieve selective binding and cytotoxicity using weak-affinity ADCs. The low binding and minimal toxicity of weak-affinity ADCs in low-Her2 cells should enhance their safety profile and mitigate against Her2-mediated adverse effects, such as hepatotoxicity noted for T-DM1 [23]. At the same time, it is recognized that T-DM1 can elicit off-target effects attributed to its payload, e.g. thrombocytopenia and elevated liver transaminases [24–28]. Nevertheless, our results support a strategy that can be applied to a large number of “dirty” targets in which target expression is present at high density on tumor tissue, yet still quite significant on normal host tissue, such as CD44.

Interestingly, the Her2 affinity range of the 7–5 and 14–13 antibody candidates (*K*_D_ range of ~70–255 nM, Table 2), chosen for SKOV3 versus MCF7 selectivity, is modestly weaker than that proposed by Slaga et al. [14], for anti-Her2/CD3 T-cell dependent bispecific (TDB) antibodies (*K*_D_ = 23–49 nM), which also rely on avidity binding to promote tumor selectivity. However, these differences in affinity may be related to the different high-Her2 density cell line models chosen (SKBr3 for the TBDs versus SKOV3), different modes of action (T-cell-mediated killing versus ADC) or the antibody formats (fusion of anti-Her2 Fab to the N-terminus of the IgG heavy chain versus conventional ADC). The affinity for an optimal ADC targeting Her2 tumors will also depend on the drug utilized. For example, a higher affinity may be required for an antibody coupled to a less potent drug such as DM1 compared to PNU, e.g. *K*_D_ in the range of the moderate variants (*K*_D_ ~15–25 nM).

Based on their weakened affinities relative to Herceptin, it is anticipated that 7–5 and/or 14–13 ADCs will provide the added benefit of improved drug delivery into solid tumors. A recent report compared the *in vivo* efficacies of 3 ADCs based on different antibodies that target tissue factor (TF) and differed in binding affinities by ~ 10–70 fold (*K*_D_ range ~ 0.5–35 nM) [29]. Treatments with the lowest-affinity (35 nM) ADC resulted in a modest improvement in pancreatic tumor (BxPC-3) reduction compared to the highest-affinity (0.5 nM) ADC. This improvement was only seen at early stages and was attributed to better ADC diffusion when tumors were small. In the case of Herceptin antibody variant 14–13 reported here, its affinity for Her2 is ~500-fold weaker than WT (*K*_D_ = 253 versus 0.5 nM, respectively) (Table 3). Hence, we expect 14–13 will be even more efficacious in terms of overcoming the binding site barrier (i.e. dissociating from Her2 at the tumor periphery) and penetrating deeply into large tumors.

In summary, we have shown that, given appropriate drug selection, exploiting avidity effects can be a powerful benefit in terms of improving tumor selectivity and widening the therapeutic index of ADCs under development.

## Materials and methods

### Cell lines

All cell lines were obtained from ATCC. SKOV3 and JIMT-1 cells were cultured in McCoy’s and DMEM media, respectively. MCF7 and Jurkat cells were cultured in RPMI 1640 media. In each case, media was supplemented with 10% fetal bovine serum (FBS).

### Antibody productions and purification

cDNAs for the designed, full-size heavy and light human IgG chains were ordered from a commercial vendor (ThermoFisher Scientific/Life Technologies Inc., Burlington, ON; GENEART, Regensburg, Germany). The antibodies were produced by co-transfection of CHO-3E7 cells. CHO-3E7 cells in were grown to a density of 2 × 10^6^ cell/mL in 150 mL of F17 medium in 1.0 L shaker flasks. The cells were transfected with 2 μg total DNA (containing 500 ng each of the heavy and light chain constructs) using PEI MAX^™^ (Polysciences, Inc., Warrington, PA). The cells were maintained at 37°C for 24 h and then fed with Tryptone N1 at 1% w/v, after which they were transferred to 32°C for 6 days. The cell culture supernatant was harvested and analyzed by SDS-PAGE for expression. Antibody was captured on MabSelect Sure column (GE Healthcare Life Sciences, Mississauga, ON), followed by elution using 100 mM Citrate, 50 mM NaCl, pH 3.0 and neutralization with 1M HEPES. Desalting and PBS exchange was achieved using ZebaSpin columns (ThermoFisher Scientific, Waltham, MA).

### Antibody quality control (QC)

Unconjugated and drug/dye-conjugated antibodies were assessed for purity and monomer status via HPLC-size exclusion chromatography (SEC) in a Superdex S200 column (GE Healthcare Life Sciences). The mobile phase was D-PBS, 0.2 M Arginine pH 7.2 run at a flow rate of 0.25 mL/min.

### Dye and drug conjugations

**pHAb conjugation**: Antibody variants were adjusted to 2 mg/mL in PBS. pHAb amine reactive dye (Promega, Madison, WI) was dissolved in DMSO at a concentration of 5 mg/mL and then added to antibody at 10:1 ratio (pHAb:antibody), followed by incubation at 25°C for 1 h. Excess dye was removed be ZebaSpin desalting column (ThermoFisher Scientific) equilibrated in PBS. The sample was centrifuged at 20,000×*g* for 10 min and supernatant containing the conjugate was retained. Dye-to-antibody ratio (DAR) was determined by OD readings at A280 and A532 nm using a NanoDrop 2000 spectrophotometer (ThermoFisher Scientific).

**AlexaFluor-488 (AF488) conjugation**: WT Herceptin was adjusted to 2 mg/mL in PBS. AF488 (Invitrogen Molecular Probes, Eugene, OR) was dissolved in N,N-Dimethyl-formamide. The conjugation reaction, purification and DAR analysis were carried out according to the manufacturer’s specifications.

**DM1 conjugation**: Primary or secondary antibody variants were combined with SMCC-DM1 (Levena Biopharma, San Diego, CA) in 1XNRC4 buffer (100 mM sodium phosphate, 20 mM NaCl, 3 mM EDTA, pH 7.4) and incubated at 25°C, 18 h. Polysorbate-20 was added to final concentration of 0.02% w/v. The reaction was passed through 3 successive ZebaSpin columns (ThermoFisher Scientific), equilibrated with 20 mM sodium-succinate, 0.02% polysorbate-20, pH 6.0. Trehalose was added to the final sample at 6% w/v. The drug-to-antibody ratio (DAR) was determined by measuring OD readings at A280 nm and A252 nm using NanoDrop 2000 and HPLC-SEC analysis.

**MMAE and PNU conjugations**: Prior to conjugation, the anti-human IgG antibody was reduced using TCEP (Tris(2-carboxyethyl)phosphine, Sigma-Aldrich, Oakville, ON) to make reactive thiols accessible. The degree of conjugation with MMAE was controlled by adjusting the molar ratio of TCEP:antibody. The reduction mixture was incubated at 37°C for 3 h with no agitation. To this was then added an 8-fold molar excess (relative to antibody) of MC-vc-PAB-MMAE (Levena Biopharma) bearing a maleimide thiol reactive group. This mixture was further incubated at 25°C for 1 h. The reaction was stopped by buffer exchange into 20 mM succinate, 0.02% w/v Polysorbate-20, 6% w/v Trehalose pH 6.0. The DAR was determined by measuring A280 nm and 248 nm. Direct conjugation of antibody variants to PNU was performed with MA-PEG4-VC-PAB-DMAE-PNU159682 (PNU) (Levena Biopharma). Each antibody (2 mg/mL) was incubated with TCEP in buffer containing 500 mM potassium phosphate, 200 mM sodium chloride, 20 mM EDTA, pH 7.2 at 37°C for 2 h. PNU was then added at 10× molar excess and incubated at 25°C for 2 h. The reaction samples were then purified via ZebaSpin columns as described above for DM1 conjugations.

### Structure-based computational design of Fab variants

The Her2-bound crystal structures of Herceptin Fab [30], and its 40-fold affinity-weakened variant bH1 [15,31], (also termed Parent 1 and Parent 2, respectively) were retrieved from the Protein Data Bank (entries 1N8Z and 3BE1, respectively). These crystal structures were used as starting points for the design of additional Fab variants with Her2 binding affinities evenly distributed within a wider range of *K*_D_ that spans several orders of magnitude. Structural preparation of crystal structures for molecular design was done as described previously [16]. Specific point-mutations were built and evaluated energetically at targeted positions within the Fab CDR loops using three programs, SIE-SCWRL [32–34], FoldX [35––36], and Rosetta [37, 38], as implemented in the ADAPT protocol [16]. A consensus score was derived for ranking the Fab mutants in terms of Her2 binding affinity relative to their respective parent Fab. This consensus scoring is based on the average Z-score over the individual raw scores calculated with the three component energy functions, SIE [33, 34], FoldX-FOLDEF [35], and Rosetta-Interface [37]. Further technical and implementation details of this consensus approach and its component methods can be found in Sulea *et al*. [39] and Vivcharuk *et al*. [16]. The FoldX-FOLDEF energy function [35], was also used to estimate the effect of substitutions on the internal stability of the Fab structure.

The final selection of the 18 Fab variants (Table 1) was based on *in silico* screening from previous ADAPT affinity maturation campaigns for Herceptin and bH1 against their Her2 antigen.[16] In these campaigns, virtual saturation mutagenesis screens in the CDRs of Herceptin and bH1 Fabs required computational evaluation of ~1200 single-point mutations in each system. Following the previously described stepwise approach underlying the ADAPT platform,[16] single mutations were selected based on *in silico* binding affinity scores and intermolecular interactions, and then sent for experimental evaluation. Single mutants validated to have the desired binding affinity trends were combined into higher-order mutants (i.e., double- and triple mutants). From the resulting pool of mutants, the set retained in Table 1 was chosen to provide even sampling within a wide range of binding affinity based on a minimal number of mutation sites.

### SPR analysis

Antibody-Her2 interactions were analysed using a Biacore T200 (GE Healthcare) surface plasmon resonance instrument. Samples were assayed at 25°C using PBS (GE Healthcare) with added 3.4 mM EDTA and 0.05% Tween 20 as running buffer. CM-5 sensorchips were prepared using the GE Healthcare anti-hFc capture kit following the recommended protocol that resulted in a 10,000 resonance unit (RU) Fc-capture surface over the sample and reference sensor surfaces. Variant antibodies were diluted to 1.25 ug/mL in running buffer and captured by flowing for 6 to 10 s at 30 μL/min to give a final Her2 binding response less than 100 RUs. The Her2 interaction was determined using Single Cycle Kinetics analysis in duplicate for each variant. High and moderate affinity variants used a 60 nM high concentration and the low affinity variants used a 180 nM high concentration, both with a 2-fold dilution series. With the high and moderate affinity variants, the flow rate of Her2 was 50 μL/min using a 180 s contact time and a 1800 s dissociation phase. The weak affinity variants had reduced contact time and flow rates to 90 s and 30 μL/min, respectively, to conserve Her2 sample. Sensorgrams were double referenced to a blank Fc-capture surface and analysed using a 1:1 binding model in BiaEvaluation software v3.1 (GE Healthcare).

### Flow cytometry saturation binding of antibodies on cells

Cells were grown in T75 flasks, and then washed twice in PBS, followed by release from plastic using Cell Dissociation buffer (Sigma) at 37°C. The suspended cells were transferred to a 50 mL conical tube and FACS buffer (D-PBS, 2% FBS) was added at a 2:1 v/v ratio (FACS buffer:cells). The cells were collected by centrifugation (233×*g*) and resuspended at 2.0 × 10^6^ cells/mL in FACS buffer at 4°C. The cells were dispensed at 1.0 × 10^5^ cells/well into 96-well v-bottom polypropylene (PP) plates (Costar, Cole-Parmer Canada Company, Montreal, CA). Pre-diluted antibody samples were then added to cells to give final volume of 100 μl/well and concentrations ranging from 0 to 200 nM or 1 μM (8-point dilution series), followed by incubation at 4°C, 2 h. The cells in the plate were then washed twice by centrifugation at 233×*g*, removal of supernatant by aspiration and cells resuspended in 200 μL FACS buffer at 4°C. The detection reagent, anti-human Fc AlexaFluor488-(Fab’)^2^ (Jackson ImmunoResearch, Westgrove, PA), was then added at a final concentration of 10 μg/mL and samples were incubated at 4°C, 1 h. The cells were washed twice in 200 μl FACS buffer, followed by addition of 120 μl 1.0% propidium iodide and samples were then transferred to Multiscreen 96-well plates (60 μm Nylon Mesh, Millipore, Etobicoke, ON) and filtered by centrifugation. The filtrate samples were collected from the Multiscreen receiver plate and transferred to a new v-bottom PP plate at 4°C. Flow cytometric analysis was performed on a BD LSR-Fortessa (BD Biosciences, San Jose, CA). The AlexaFluor488 fluorescence was measured using the 488 nm laser as an excitation source and the 530/30nm band pass filter. The median fluorescence intensity (MFI) was determined for 3000 alive cells per sample (Gating strategy: All cells/Singlets/Alive cells (PI negative)) using BD FACSDiva software (BD Biosciences).

### Flow cytometry competition binding

Binding competition of Herceptin antibody variants with AF488-labelled WT Herceptin was performed using SKOV3 cells. The procedure was similar to that described above, except a secondary detection antibody was not used and diluted antibody samples were added 1:1 to 40 nM AF488-Herceptin prior to cell binding at 4°C, 2 h.

### Internalization assays and high content imaging

**Indirect internalization assay**: SKOV3 cells were seeded in 96-well plates (Corning, Fisher Scientific, Toronto, ON) at 5 × 10^3^ cells/well and incubated at 37°C, 18 h. Antibody variants were added to cell media and combined with a proprietary secondary anti-huIgG1-pHAb (molar ratio Ab:secondary = 1:1) and incubated at RT, 30 min. The antibody samples were added to cells in an 8-point dilution series, followed by incubation at 37°C, 18 h. The cells were washed thrice with D-PBS (with calcium and magnesium) (Wisent, St-Bruno, QC) at RT and then treated with Hoechst 33342 stain (10 μg/mL, Invitrogen Molecular Probes) at 37°C, 15 min. Imaging was performed using ImageXpress Micro XLS (Molecular Devices, San Jose, CA) with the following channels and exposure times; TRITC (250 ms) and DAPI (70 ms) and 20× objective. The images were analyzed using the Transfluor module. Internalization was measured as integrated Fluorescence Index (FI)/nucleus.

**Direct internalization assay**: SKOV3 or JIMT-1 cells were seeded onto 384-well plates (Corning #3721) at 2.5 × 10^3^ and 3.0 × 10^3^ cells/well, respectively, and incubated at 37°C, 18 h. pHAb-labelled antibody variants were added to cells in an 8-point dilutions series, followed by incubation at 37°C, 18 h. The cells were treated with Hoechst stain at 37°C, 15 min and then washed twice with D-PBS (with calcium and magnesium). Images were captured in ImageXpress with the following settings: SKOV3 cells; TRITC (100 ms) and DAPI (25 ms), JIMT-1 cells; TRITC (300 ms) and DAPI (25 ms).

### ADC cytotoxicity assays

Antibody variants were tested for their effects on viability on Her2 cell lines, including SKOV3, JIMT-1, MCF7 and Her2-negative Jurkat cells. Cells were seeded at 200, 500, 150 and 1500 cells/well for the four cell lines respectively in 384-well plates (Corning^®^ 384 Well White Flat Bottom Polystyrene TC-Treated Microplates, Cat. # 3570). For the indirect ADC screens, cells were allowed to grow for five days in the presence of serial dilutions of the test antibodies or benchmark controls ranging from 100 nM to 0.0017 nM, combined with an excess of proprietary anti-human IgG-SMCC-DM1 or IgG-vcMMAE. For direct ADC assays, SKOV3 cells were grown for five days in the presence of serial dilutions of DM1- or PNU-conjugated test antibodies ranging from 100 nM to 0.0017 nM. After five days (37°C, 5% CO2, humidified incubator), the number of viable cells was determined using CellTiterGlo (Promega, Madison, WI), based on quantitation of the ATP present in each well, which signals the presence of metabolically active cells. Signal output was measured on a luminescence plate reader (Envision, Perkin Elmer, Shelton, CT) set at an integration time of 0.1 s. Integration time is adjusted to minimize signal saturation at high ATP concentration.

**Data analysis**: The signal value in each well (S) is normalized to the negative control wells (cells without drug) (NC) and expressed as % Surviving Fraction.

%SurvivingFraction=(S×100)/NC

Dose-response curves of % Survival Fraction vs. Log_10_ of concentration were fit using GraphPad Prism 6.0 with a four-parameter logistic model to estimate IC_50_ and maximum efficacy.

Y=Bottom+(Top−Bottom)/(1+10^((LogIC50−X)×HillSlope))

IC_50_ is the concentration of agonist that gives a response half way between Bottom and Top. HillSlope describes the steepness of the curves. Top and Bottom are plateaus in the units of the Y axis.

## Supporting information

S1 Fig HPLC-SEC QC profiles (left panels) for selected antibody variants and confirmed monomer purity and recovery from the SEC matrix (right table). (TIF)

S2 Fig Saturation binding apparent *K*_D_s and *B*_max_ values for antibody variants on MCF7 and SKOV3 cells, as determined by flow cytometry. **(A, B)** MCF7 cells. **(C, D)** SKOV3 cells. MFI = median fluorescence intensity.
(TIF)

S3 Fig Saturation binding *B*_max_ values of selected antibody variants on cells as determined by flow cytometry. **(A)** MCF7 and **(B)** SKOV3 cells.
(TIF)

S4 Fig Competition binding curves and IC_50_s of antibody variants against AF488-labelled WT antibody in SKOV3 cells. (TIF)

S5 Fig Indirect and direct internalization of antibody variants in low-Her2 MCF7 cells. Shown are fluorescence intensity (FI) values measured by ImageXpress high-content imaging for cells treated with **(A)** variants plus pHAb-labelled detection antibody or **(B)** pHAb-conjugated variants at 37°C for 18 h. The fluorescence values are for 4 incremental antibody concentrations in the lower dose range of the plate assays, as indicated. In contrast to WT (2–1), the values for moderate (2–5) and weak variants (14–13, 7–5, 16–13) were not sufficiently above Synagis background (dotted lines) and/or too variable with these cells. Therefore it was not possible to generate full-dose curves or EC50s.
(TIF)

S6 Fig Indirect DM1 ADC cytotoxicity of antibody variants in Her2-negative Jurkat cells. DM1 = unconjugated DM1.
(TIF)

S7 Fig Indirect DM1 ADC cytotoxicity screens of antibody variants in high-Her2 (SKOV3) and low-Her2 (JIMT-1 and MCF7) cell lines. Shown are representative growth-inhibition curves for **(A)** SKOV3, **(B)** JIMT-1 and **(C)** MCF7 cells, following treatment for 5 days at 37°C with the indicated antibody variants combined with DM1-secondary antibody. T-DM1 = WT Herceptin-DM1 control.
(TIF)

S8 Fig Indirect ADC cytotoxicity IC_50_s derived from DM1 (panels A-C) and MMAE ADC (panels D-F) screens. The indicated cells were treated for 5 days at 37°C with antibody variants combined with DM1 or MMAE conjugated secondary antibody. For the Synagis antibody control with MMAE in JIMT-1 and MCF7 cells, no toxicity was observed and hence IC_50_s were not determined (ND).
(TIF)

S9 Fig Growth inhibition curves for SKOV3, JIMT-1 and MCF7 cells treated with DM1-conjugated antibodies. The cells were treated with DM1 ADCs for 5 days at 37°C. The data are normalized, based on DAR (Drug Antibody Ratio), to account for DAR differences between antibody variants. T-DM1 = WT Herceptin-DM1 control.
(TIF)

S10 Fig Binding of selected Herceptin variant Fabs to SKOV3 cells as determined by flow cytometry. (TIF)

S11 Fig Direct ADC cytotoxicity of MMAE conjugated variants in SKOV3 cells. Shown are *s*urvival curves for SKOV3 cells treated with the indicated antibody-MMAE variants or Synagis-MMAE. Percent survival values for cells treated with 6 and 100 nM of unconjugated MMAE drug are shown for comparison.
(TIF)

S1 Table Efficiency of direct and indirect internalizations. (DOCX)

S2 Table Quality control of pHAb-conjugated antibodies. (DOCX)

S3 Table SPR quality control of DM1-conjugated antibody variants. (DOCX)
