# Supplementary figures and images for: Binding and functional profiling of antibody mutants guides selection of optimal candidates as antibody drug conjugates
Source: PLoS One. 2019 Dec 31;14(12):e0226593. doi: 10.1371/journal.pone.0226593 (PMC6938348; doi:10.1371/journal.pone.0226593)

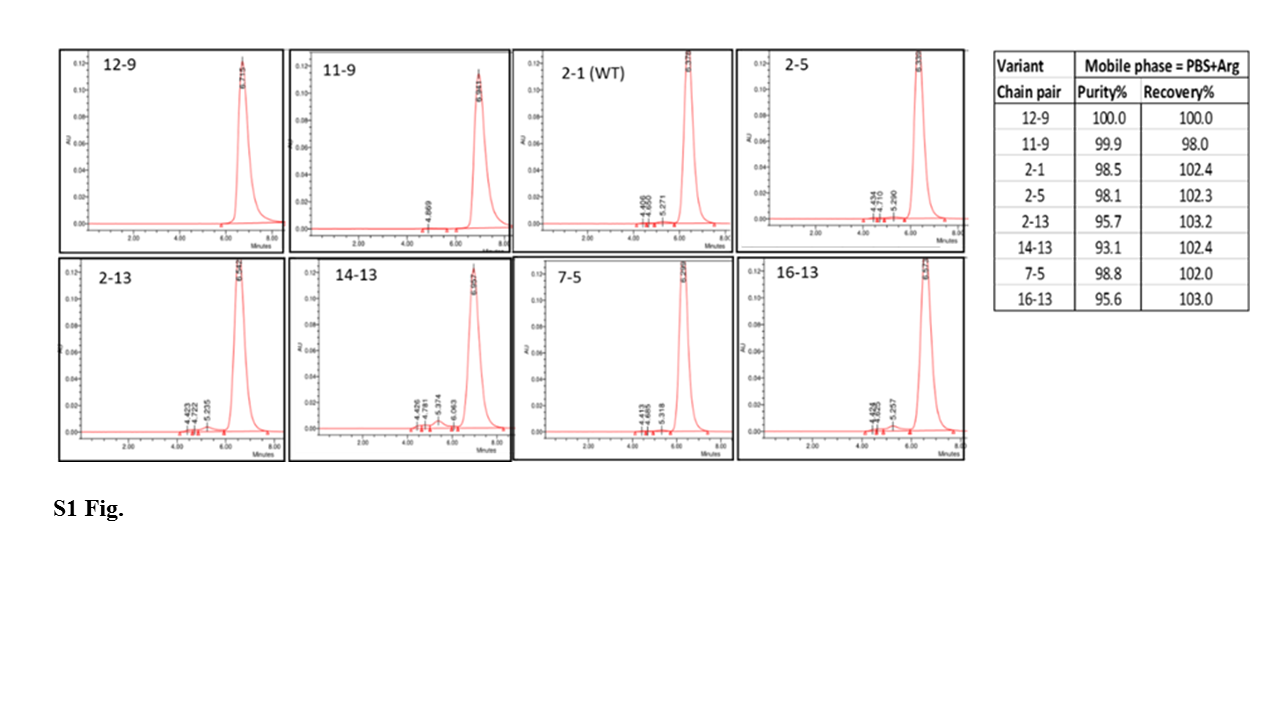

Supplement: S1 Fig — (TIF) [file pone.0226593.s001.tif]

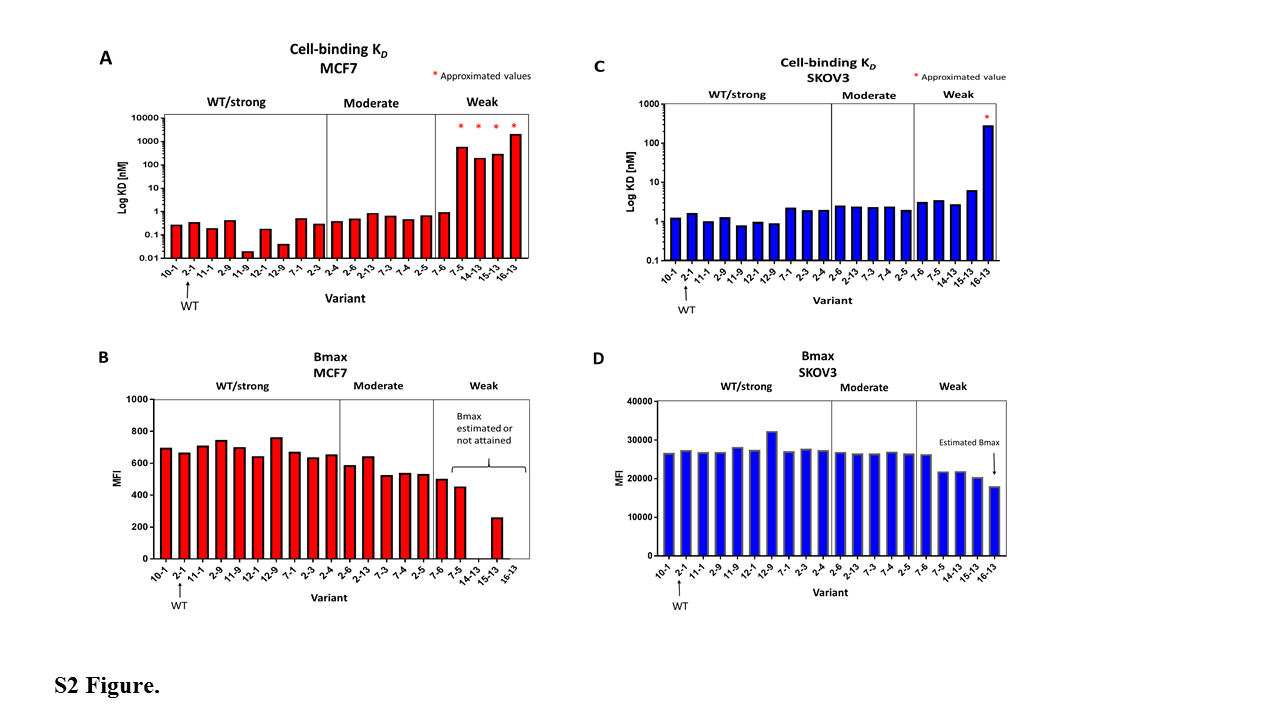

Supplement: S2 Fig — (A, B) MCF7 cells. (C, D) SKOV3 cells. MFI = median fluorescence intensity. (TIF) [file pone.0226593.s002.tif]

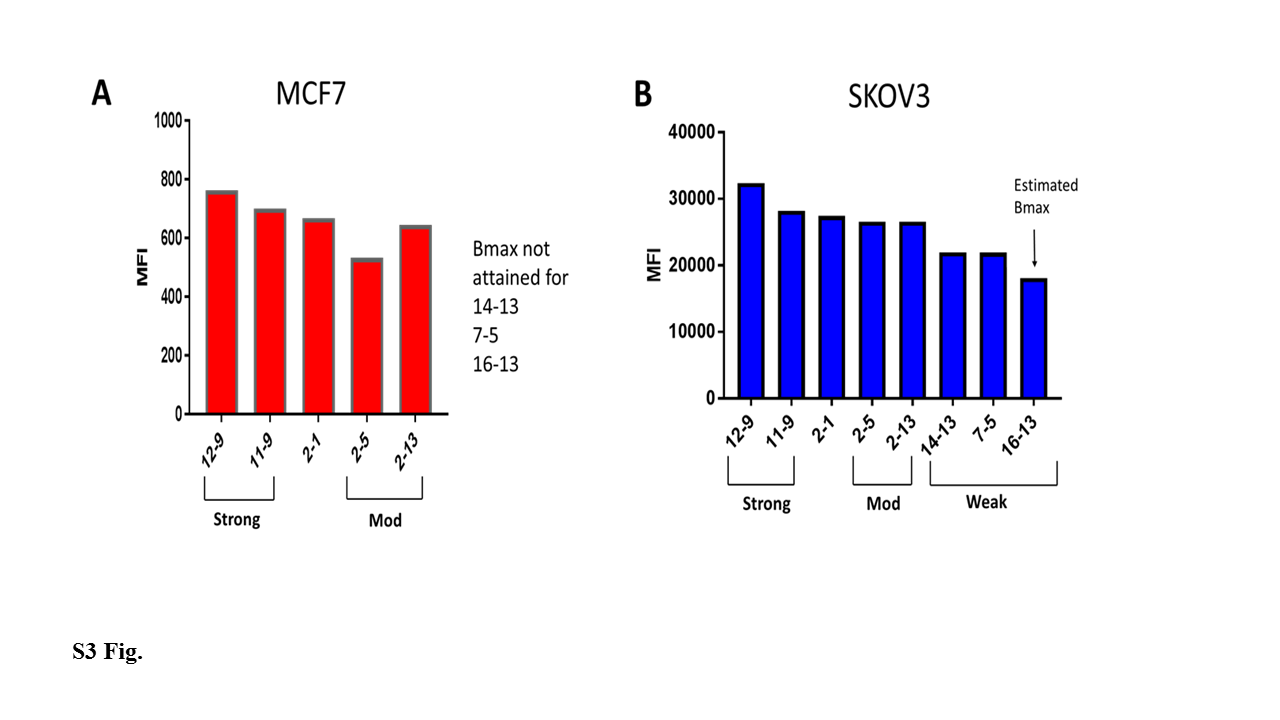

Supplement: S3 Fig — (A) MCF7 and (B) SKOV3 cells. (TIF) [file pone.0226593.s003.tif]

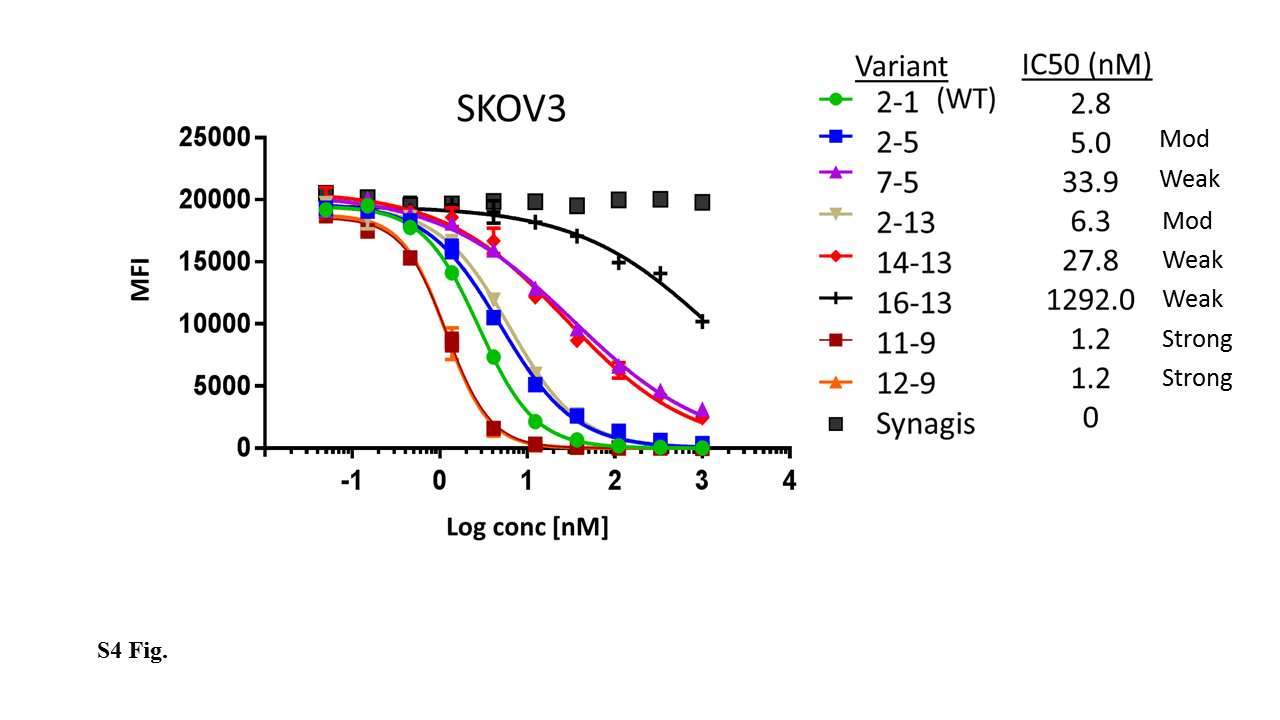

Supplement: S4 Fig — (TIF) [file pone.0226593.s004.tif]

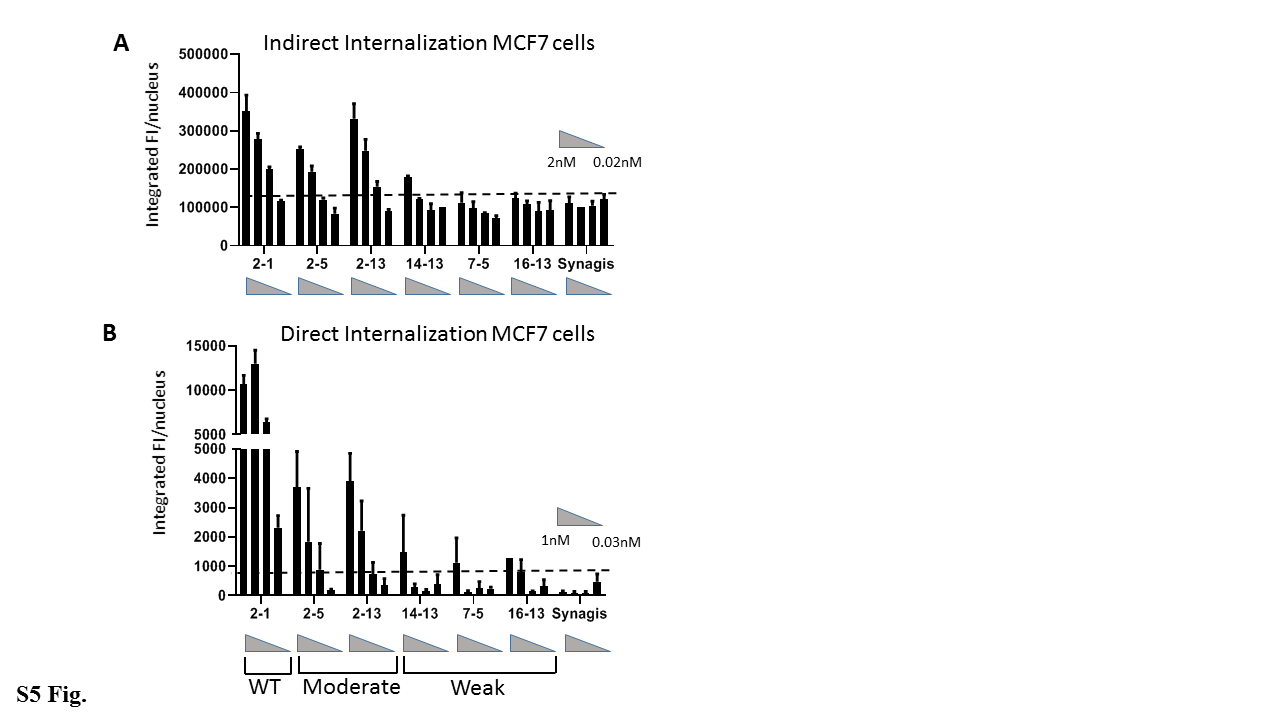

Supplement: S5 Fig — Shown are fluorescence intensity (FI) values measured by ImageXpress high-content imaging for cells treated with (A) variants plus pHAb-labelled detection antibody or (B) pHAb-conjugated variants at 37°C for 18 h. The fluorescence values are for 4 incremental antibody concentrations in the lower dose range of the plate assays, as indicated. In contrast to WT (2–1), the values for moderate (2–5) and weak variants (14–13, 7–5, 16–13) were not sufficiently above Synagis background (dotted lines) and/or too variable with these cells. Therefore it was not possible to generate full-dose curves or EC50s. (TIF) [file pone.0226593.s005.tif]

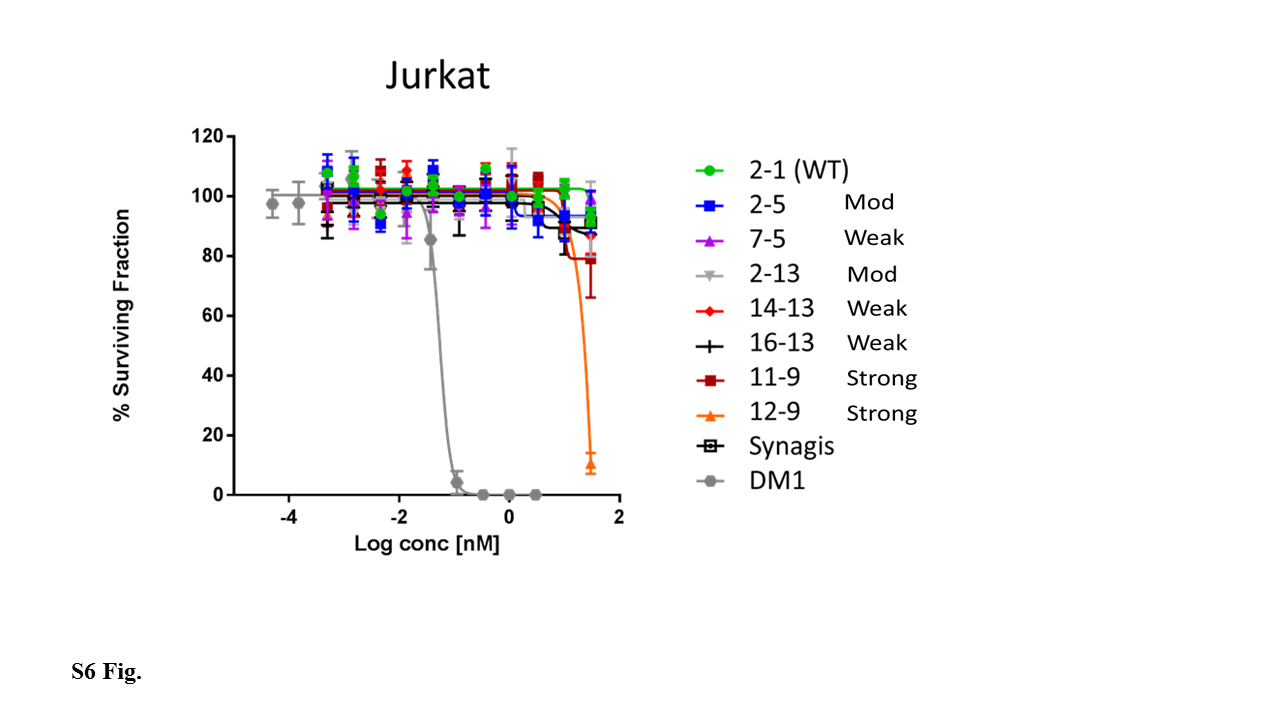

Supplement: S6 Fig — DM1 = unconjugated DM1. (TIF) [file pone.0226593.s006.tif]

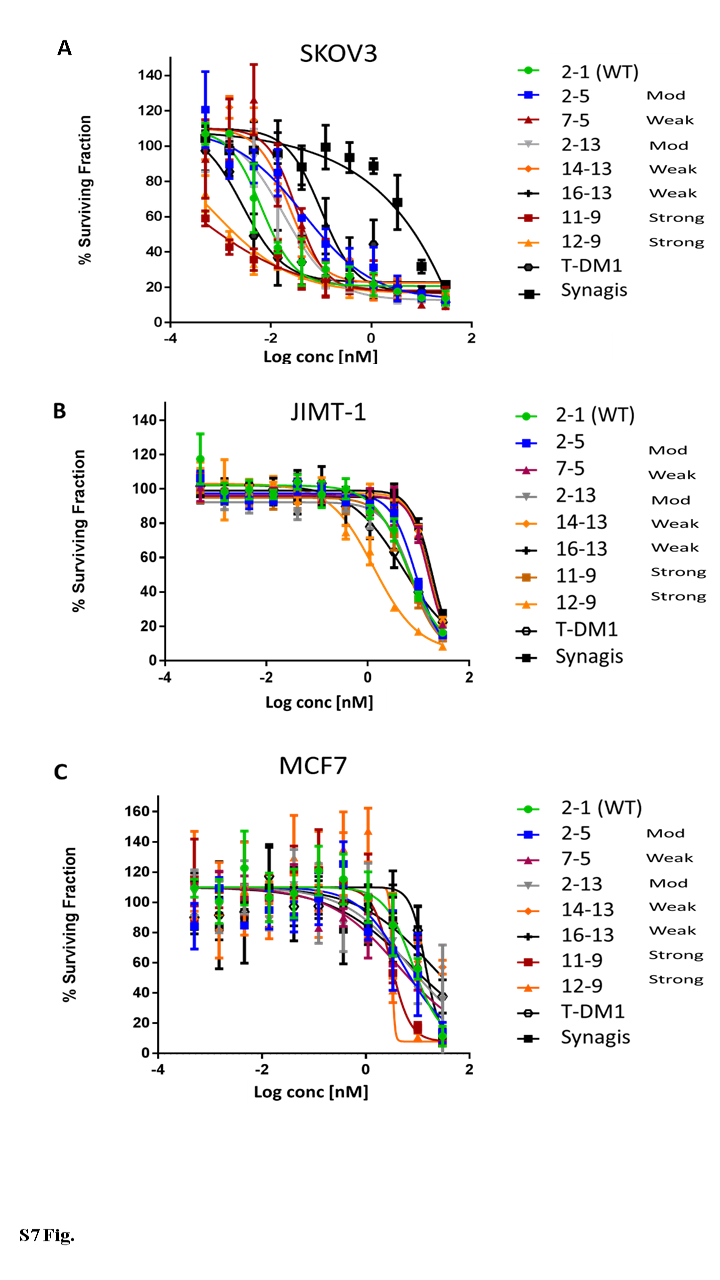

Supplement: S7 Fig — Shown are representative growth-inhibition curves for (A) SKOV3, (B) JIMT-1 and (C) MCF7 cells, following treatment for 5 days at 37°C with the indicated antibody variants combined with DM1-secondary antibody. T-DM1 = WT Herceptin-DM1 control. (TIF) [file pone.0226593.s007.tif]

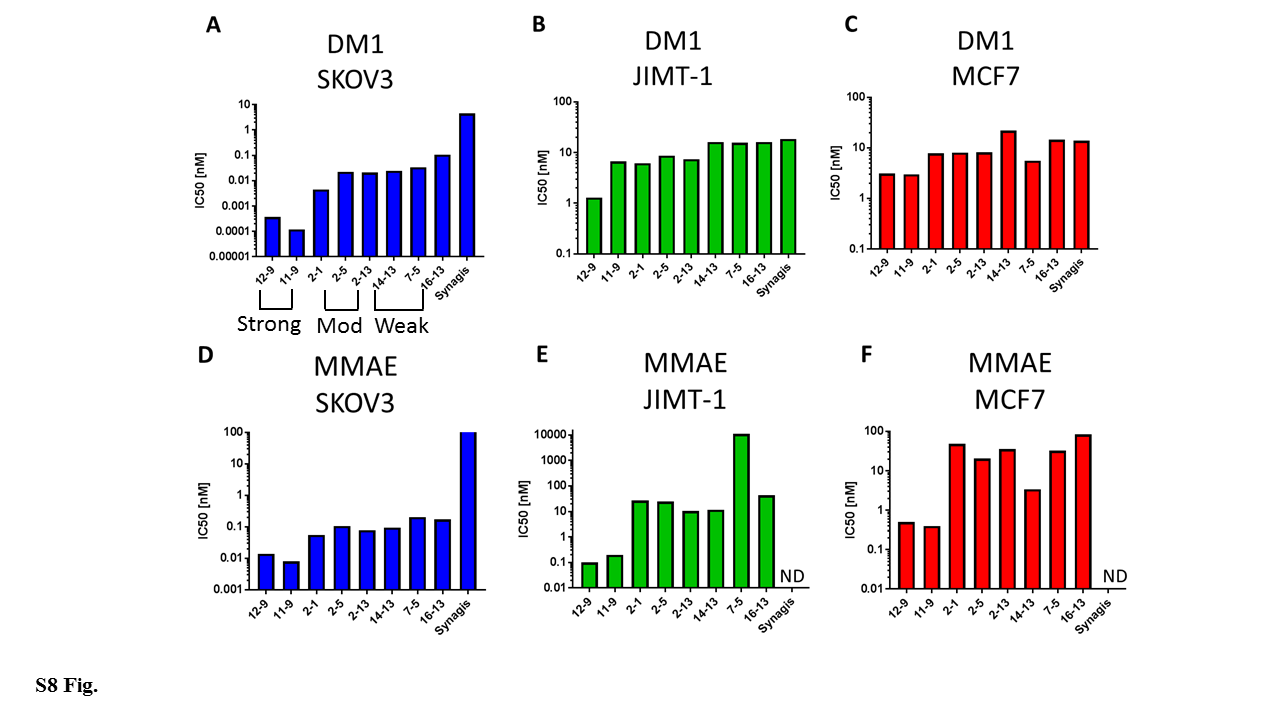

Supplement: S8 Fig — The indicated cells were treated for 5 days at 37°C with antibody variants combined with DM1 or MMAE conjugated secondary antibody. For the Synagis antibody control with MMAE in JIMT-1 and MCF7 cells, no toxicity was observed and hence IC50s were not determined (ND). (TIF) [file pone.0226593.s008.tif]

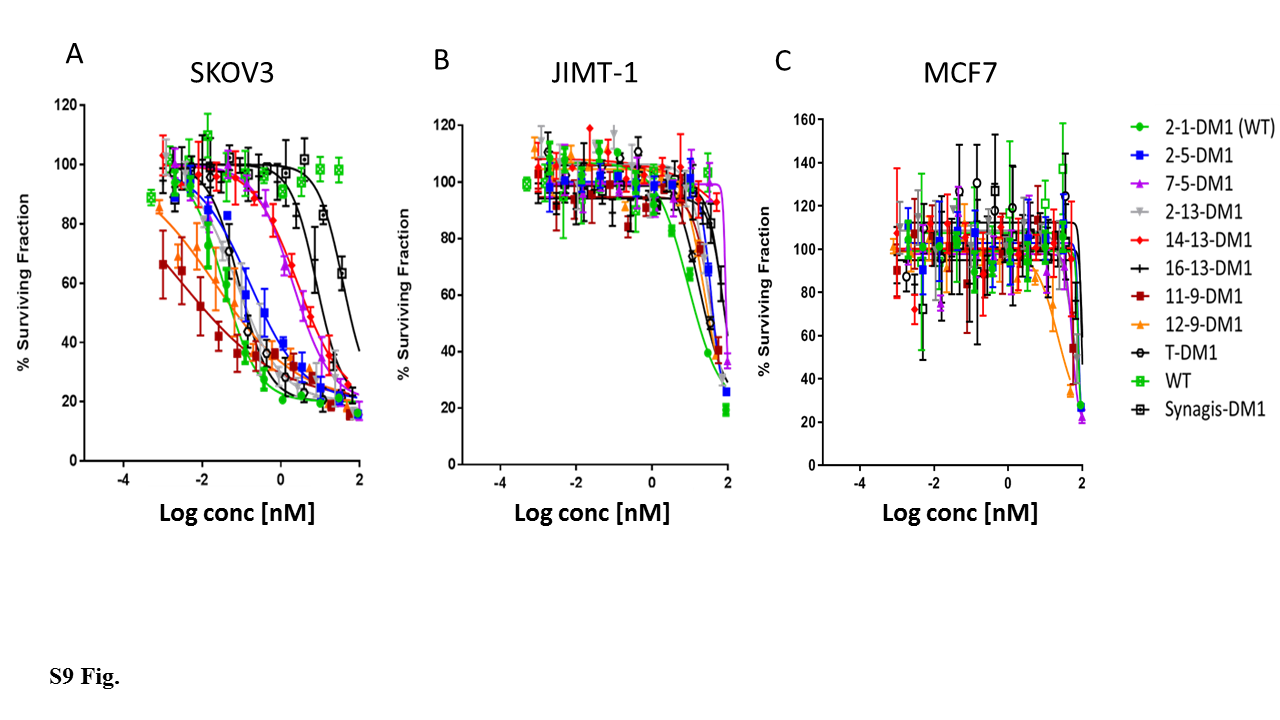

Supplement: S9 Fig — The cells were treated with DM1 ADCs for 5 days at 37°C. The data are normalized, based on DAR (Drug Antibody Ratio), to account for DAR differences between antibody variants. T-DM1 = WT Herceptin-DM1 control. (TIF) [file pone.0226593.s009.tif]

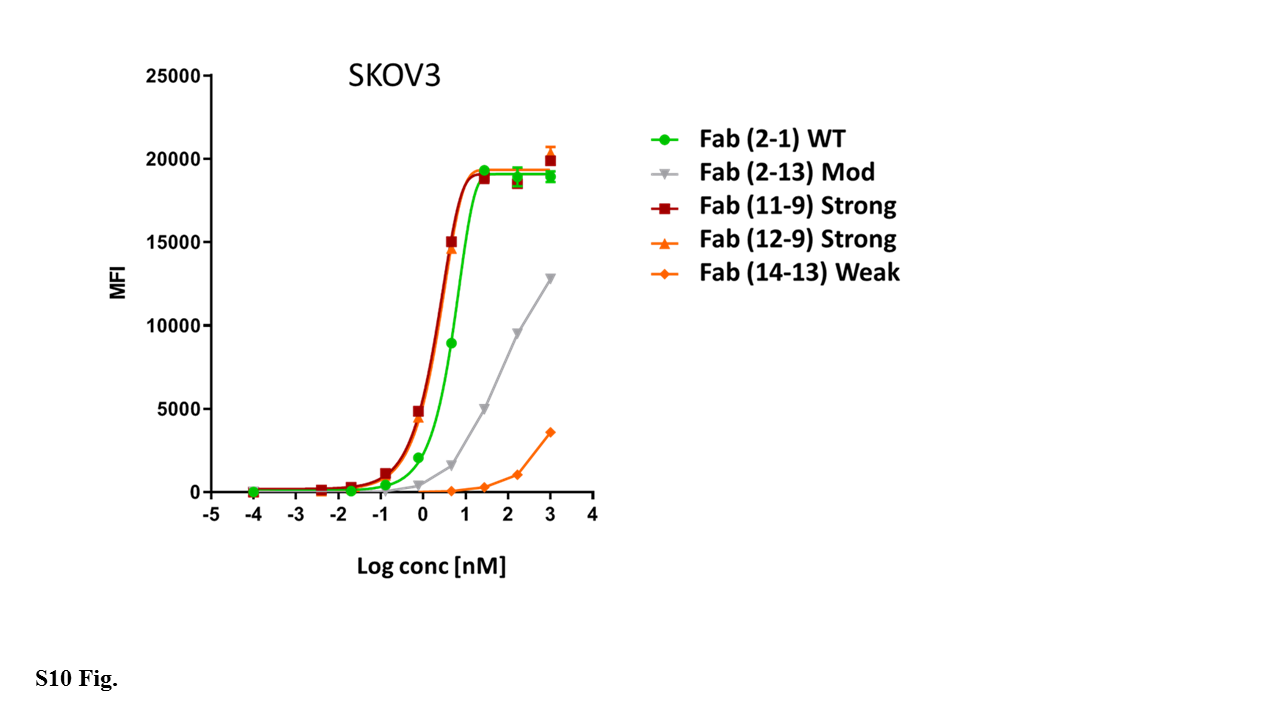

Supplement: S10 Fig — (TIF) [file pone.0226593.s010.tif]

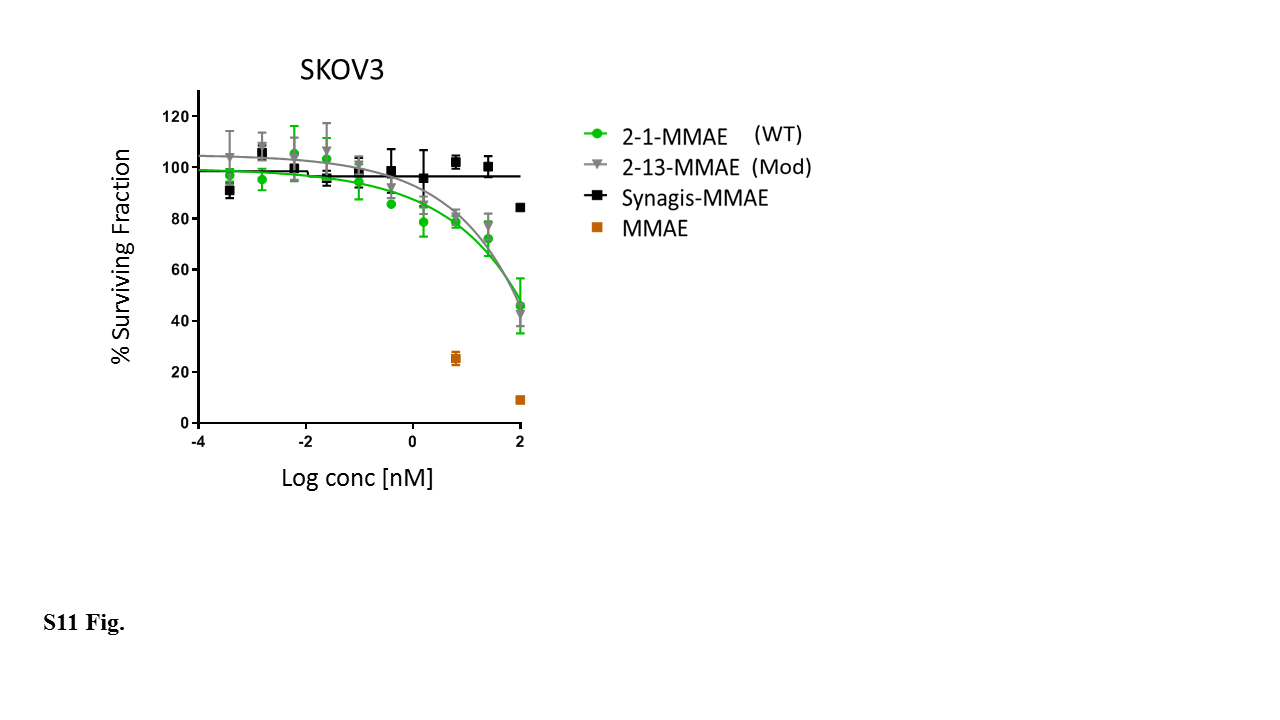

Supplement: S11 Fig — Shown are survival curves for SKOV3 cells treated with the indicated antibody-MMAE variants or Synagis-MMAE. Percent survival values for cells treated with 6 and 100 nM of unconjugated MMAE drug are shown for comparison. (TIF) [file pone.0226593.s011.tif]
