# Supplementary material for: Binding and functional profiling of antibody mutants guides selection of optimal candidates as antibody drug conjugates
Source: PLoS One. 2019 Dec 31;14(12):e0226593. doi: 10.1371/journal.pone.0226593 (PMC6938348; doi:10.1371/journal.pone.0226593)
Supplement: S1 Table — (DOCX) [file pone.0226593.s012.docx]

**S1 Table .** **Efficiency of direct and indirect internalizations.**

| **FSA class** | **Variant** | **Indirect** ^a^ |  | **Direct** ^b^ | |
| --- | --- | --- | --- | --- | --- |
|  |  | **SKOV3** |  | **SKOV3** | **JIMT1** |
|  |  | **EC_50_** [nM] |  | **EC_50_**  [nM] | **EC_50_**  [nM] |
| Strong | 12-9 | 0.41 |  | 0.41 | 0.07 |
| Strong | 11-9 | 0.44 |  | 0.32 | 0.07 |
| WT | 2-1 | 0.55 |  | 0.32 | 0.21 |
| Moderate | 2-5 | 1.08 |  | 1.45 | 25.09 |
| Moderate | 2-13 | 0.65 |  | 1.35 | 4.63 |
| Weak | 14-13 | 3.75 |  | 59.03 | 0 |
| Weak | 7-5 | 1.99 |  | 19.22 | 0 |
| Weak | 16-13 | 5.10 |  | 74.89 | 0 |
|  | Synagis | 0 |  | 0 | 0 |

^a^ Internalization using antibody variant + pHAb-secondary antibody.

^b^ Internalization using pHAb-conjugated antibody variant.
