# Supplementary material for: Binding and functional profiling of antibody mutants guides selection of optimal candidates as antibody drug conjugates
Source: PLoS One. 2019 Dec 31;14(12):e0226593. doi: 10.1371/journal.pone.0226593 (PMC6938348; doi:10.1371/journal.pone.0226593)
Supplement: S2 Table — (DOCX) [file pone.0226593.s013.docx]

**S2 Table.** Quality control of pHAb-conjugated antibodies

| **FSA class** | **Variant** | **DAR^a^** | **% Monomer^b^** |
| --- | --- | --- | --- |
| Strong | 12-9-pHAb | 1.4 | 94.7 |
| Strong | 11-9-pHAb | 1.8 | 93.9 |
| WT | 2-1-pHAb | 1.8 | 95.5 |
| Moderate | 2-5-pHAb | 1.9 | 95.4 |
| Moderate | 2-13-pHAb | 1.9 | 93.1 |
| Weak | 14-13-pHAb | 1.7 | 93.3 |
| Weak | 7-5-pHAb | 1.9 | 97.3 |
| Weak | 16-13-pHAb | 1.8 | 93.9 |
|  | Synagis-pHAb | 2.3 | 96.3 |

^a^DAR determined via NanoDrop 2000

^b^Monomer (non-aggregate) content determined via HPLC-SEC
