# Supplementary material for: Binding and functional profiling of antibody mutants guides selection of optimal candidates as antibody drug conjugates
Source: PLoS One. 2019 Dec 31;14(12):e0226593. doi: 10.1371/journal.pone.0226593 (PMC6938348; doi:10.1371/journal.pone.0226593)
Supplement: S3 Table — (DOCX) [file pone.0226593.s014.docx]

**S3 Table.** SPR quality control of DM1-conjugated antibody variants.

| **Variant** | **FSA class** | **% Theoretical maximum Her2 bound ^a^**  **Average (n=2)** | **% Her2 bound relative to WT ^b^ Average (n=2)** |
| --- | --- | --- | --- |
| 2-1-DM1 | WT | 57.2 | 85.4 |
| 11-9-DM1 | Strong | 29.5 | 44.1 |
| 12-9-DM1 | Strong | 20.5 | 30.6 |
| 2-1 | WT | 65.9 | 100 |

^a^ Percent of theoretical maximum Her2 binding based on the ratio of calculated maximum response from sensorgram fits (Calculated Rmax) over theoretical maximum response if captured antibody surface was 100% active (Theoretical Rmax).

% Theoretical Rmax = ((Calculated Rmax)/(Theoretical Rmax))*100.

Theoretical Rmax = ((MW Her2-ED * 2 binding sites)/(MW antibody variant))*RUs antibody capture, where MW is the molecular weight and RUs are SPR response units.

^b^ Amount of the % Theoretical maximum Her2 bound relative to unconjugated parental variant 2-1.
